# Synthesis, DFT Study, and In Vitro Evaluation of Antioxidant Properties and Cytotoxic and Cytoprotective Effects of New Hydrazones on SH-SY5Y Neuroblastoma Cell Lines

**DOI:** 10.3390/ph16091198

**Published:** 2023-08-23

**Authors:** Diana Tzankova, Hristina Kuteva, Emilio Mateev, Denitsa Stefanova, Alime Dzhemadan, Yordan Yordanov, Alexandrina Mateeva, Virginia Tzankova, Magdalena Kondeva-Burdina, Alexander Zlatkov, Maya Georgieva

**Affiliations:** 1 Department “Pharmaceutical Chemistry”, Faculty of Pharmacy, Medical University-Sofia, 2 Dunav Str., 1000 Sofia, Bulgaria; d.tsankova@pharmfac.mu-sofia.bg (D.T.); e.mateev@pharmfac.mu-sofia.bg (E.M.); a.dineva@pharmfac.mu-sofia.bg (A.M.); azlatkov@pharmfac.mu-sofia.bg (A.Z.); 2 Laboratory “Drug metabolism and Drug Toxicity”, Department “Pharmacology, Pharmacotherapy and Toxicology”, Faculty of Pharmacy, Medical University-Sofia, 2 Dunav Str., 1000 Sofia, Bulgaria; hristina.kuteva@abv.bg (H.K.); denitsa.stefanova@pharmfac.mu-sofia.bg (D.S.); yyordanov@pharmfac.mu-sofia.bg (Y.Y.); vtzankova@pharmfac.mu-sofia.bg (V.T.); mkondeva@pharmfac.mu-sofia.bg (M.K.-B.)

**Keywords:** pyrrole, synthesis, DFT, antioxidant, SH-SY5Y, cellular toxicity, cell protection

## Abstract

A series of ten new hydrazide–hydrazone derivatives bearing a pyrrole ring were synthesized and structurally elucidated through appropriate spectral characteristics. The target hydrazones were assessed for radical scavenging activity through 1,1-diphenyl-2-picrylhydrazyl (DPPH) and 2,2′-azino-bis(3-ethylbenzothiazoline-6-sulphonic acid) (ABTS) tests, with ethyl 5-(4-bromophenyl)-1-(2-(2-(4-hydroxy-3,5-dimethoxybenzylidene)hydrazine-yl)-2-oxoethyl)-2-methyl-1H-pyrrole-3-carboxylate (**7d**) and ethyl 5-(4-bromophenyl)-1-(3-(2-(4-hydroxy-3,5-dimethoxybenzylidene) hydra zine-yl)-3-oxopropyl)-2-methyl-1H-pyrrole-3-carboxylate (**8d**) highlighted as the best radical scavengers from the series. Additional density functional theory (DFT) studies have indicated that the best radical scavenging ligands in the newly synthesized molecules are stable, do not decompose into elements, are less polarizable, and with a hard nature. The energy of the highest occupied molecular orbital (HOMO) revealed that both compounds possess good electron donation capacities. Overall, **7d** and **8d** can readily scavenge free radicals in biological systems via the donation of hydrogen atoms and single electron transfer. The performed in vitro assessment of the compound’s protective activity on the H_2_O_2_-induced oxidative stress model on human neuroblastoma cell line SH-SY5Y determined **7d** as the most perspective representative with the lowest cellular toxicity and the highest protection.

## 1. Introduction

The generation of reactive oxygen species (ROS) leads to oxidative damage to biomolecules, such as cellular proteins, lipids, and DNA, and, as a result, to disruption to normal cellular functions [1]. A gradual decline in cellular antioxidant defense mechanisms due to the generation of ROS during aging leads to increases in oxidative stress. Therefore, aging, various genetic mutations, the influence of the environment, and the associated increase in oxidative stress are a prerequisite for the development of many neurodegenerative diseases, including Parkinson’s disease (PD), Alzheimer’s disease (AD), Huntington’s disease (HD), amyotrophic lateral sclerosis (ALS), and many others [2]. Accordingly, the discovery of novel active substances with antioxidant potential is the basis of many programs for providing effective neuroprotection in the therapy of neurodegenerative diseases [3].

A lot of market drugs containing pyrrole ring show different pharmacological activities, such as antipsychotics [4], antidepressants [5], anticonvulsants [6], anti-inflammatories [7], and many others. There is increased interest in pyrroles and pyrrole derivatives exhibiting proven antioxidant effects [8,9]. Many heterocyclic hydrazones have shown antioxidant and neuroprotective activity, too [10].

For example, Boulebd et al. [11] successfully synthesized a series of new phenolic hydrazide-hydrazone derivatives and tested them for radical scavenging activity using DPPH and ABTS methods. The effects were compared with the action of ascorbic acid and Trolox, compounds with proven antioxidant activity. It was found that the synthesized new derivatives exhibited antioxidant activity comparable to those of ascorbic acid and Trolox [11]. In another study [12], twelve heterocyclic compounds bearing hydrazone functional groups were evaluated for antioxidant activity using DPPH, ABTS, and DMSO alkaline assays. The results showed that these heterocyclic compounds are potent antiradical agents [12]. In addition, a preliminary study performed by Tzankova et al. [13] identified the promising radical scavenging potential of pyrrole-based hydrazones, as determined through DPPH and ABTS assays, which pointed our attention toward the synthesis of analogous representatives in an attempt to enrich the variety of molecules.

**Figure 1:**
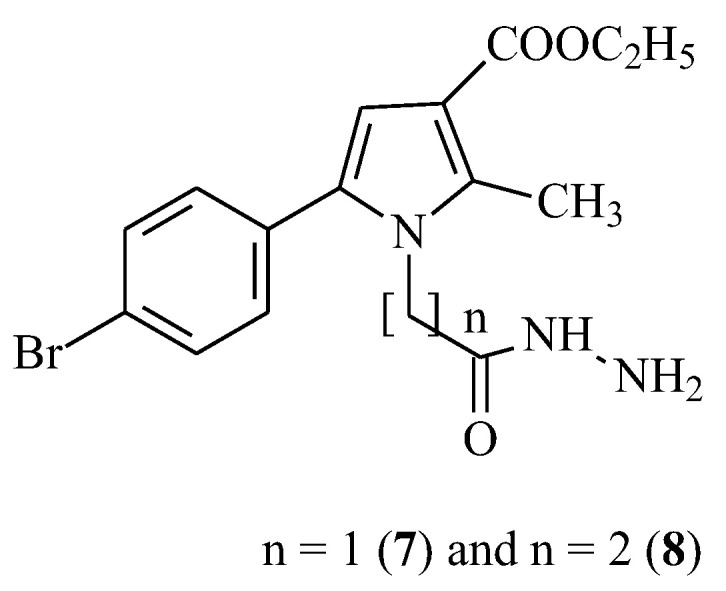
General structure of the initial pyrrole hydrazides.

In the current study, the initial hydrazides were synthesized in our laboratory, as previously explained in detail by Bijev et al. [14] for hydrazide **7** and Georgieva et al. [15] for hydrazide **8**, with the general structure presented in Figure 1.

The following study is focused on the synthesis of new hydrazones containing a pyrrole ring system and the evaluation of their in vitro safety profile on human neuroblastoma SH-SY5Y cells, radical scavenging activity through 1,1-diphenyl-2-picrylhydrazyl (DPPH) and 2,2′-azino-bis(3-ethylbenzothiazoline-6-sulphonic acid) (ABTS) assays and in silico assessment of possible antioxidant mechanisms through DFT calculations. The most promising structures are evaluated for antioxidative protective properties in a H_2_O_2_-induced oxidative stress model on SH-SY5Y cells.

## 2. Results

### 2.1. Chemistry

#### 2.1.1. Synthesis of the N-pyrrolyl hydrazides 7 (ethyl 5-(4-bromophenyl)-1-(2-hydrazinyl-2-oxoethyl)-2-methyl-1H-pyrrole-3-carboxylate) and 8 (ethyl 5-(4-bromophenyl)-1-(3-hydrazinyl-3-oxopropyl)-2-methyl-1H-pyrrole-3-carboxylate)

**Scheme 1:**
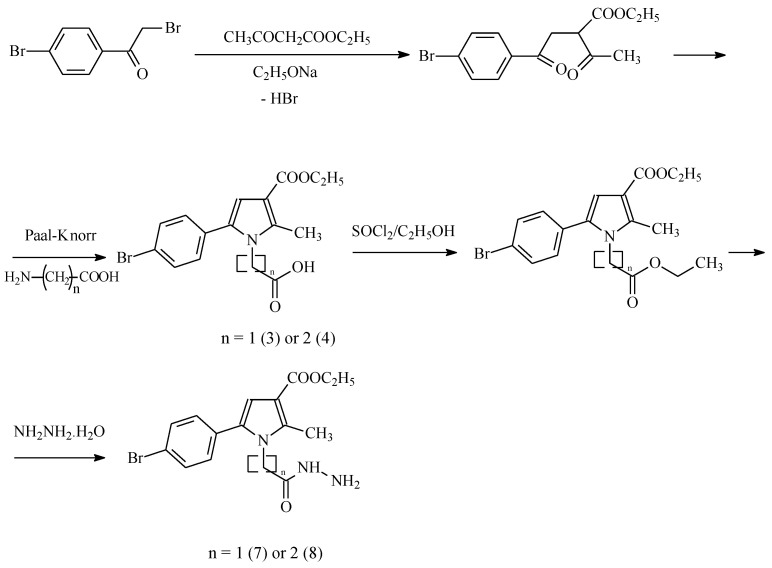
Synthesis of the initial hydrazides **7** and **8** [14,15].

The pyrrole ring in the target hydrazone molecules was formed through a Paal–Knorr cyclization reaction based on the C-alkylation of a 1,3-dicarbonyl compound to the corresponding 1,4-dicarbonyl derivative (**2**), cycled afterward with the corresponding amino acids L-glycine (n = 1) and L- *β*-alanine (n = 2) to give the following N-substituted pyrrole carboxylic acids (**3** and **4**, respectively). An esterification of the obtained acids was conducted, followed by a hydrazinolysis reaction with hydrazine hydrate, to give the target hydrazides (**7** and **8**, respectively) according to the procedure presented in Scheme 1 and described in detail in [14,15].

#### 2.1.2. Synthesis of the New N–pyrrolylhydrazide–hydrazones **7a**–**e** and **8a**–**e**

**Scheme 2:**
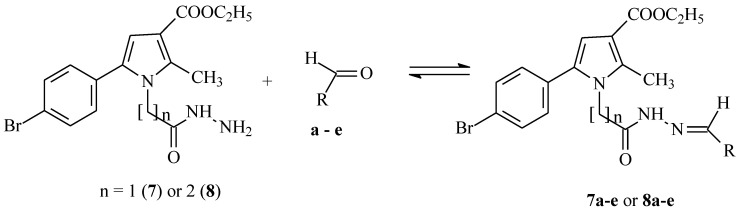
Synthesis of the new hydrazone compounds.

**Figure 2:**
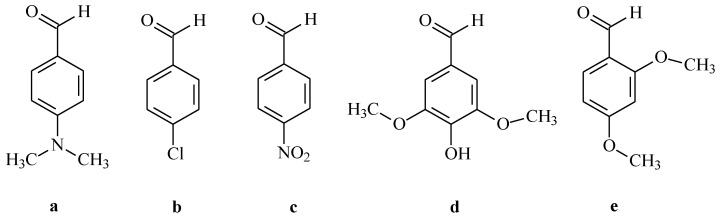
Used aldehydes (**a–e)**.

The novel series of N–pyrrolylhydrazide–hydrazone derivatives were prepared under microsynthesis scale conditions through condensation reaction from the previously synthesized in our laboratory hydrazides **7** and **8** and the selected carbonyl partners (Figure 2), assuring about 64–86% yield of the purified product. The new compounds were synthesized according to the procedure presented in Scheme 2.

The reaction conditions were altered, as presented in Table 1, wherein the reflux in glacial acetic acid was selected as the most appropriate. All the new derivatives were obtained according to that presented in the Materials and Methods section procedures.

The synthesized pyrrole hydrazones are stable at room temperature for long periods of time. The structures of the new compounds were elucidated by melting points, TLC characteristics, IR, and ^1^H-NMR spectral data, followed by MS data. The results from the spectral analysis confirmed that the new compounds are consistent with the expected structure. The corresponding experimental IR, ^1^H-NMR, and LC-MS spectra of the target derivatives are supplied as Supplementary Materials. The purity of the obtained compounds was proven by corresponding elemental analyses.

The IR data determine the presence of new signals at 3245 cm^−1^ for the valence asymmetric (ν^as^) vibrations for the amide NH group in the molecule of the new hydrazones and a band at 1666 cm^−1^ (Amide I) and 1533 cm^−1^ for deformational (*δ*) vibrations of the amide NH group (Amide II). In addition, the appearance of a band at around 810 cm^−1^ determines the presence of *p*-substituted phenyl residue. The ester group (COOC_2_H_5_) in the third position in the central pyrrole ring is pointed by the appearance of a band at around 1693–1698 cm^−1^. A confirmation of the structural elucidation may be seen in the relevant ^1^H NMR spectra, where the corresponding groups are available at 7.86 ppm for the CH=N group and at 11.38 ppm for the CONH group, respectively. The obtained values from the experimental spectral data are consistent with the theoretical ones.

The corresponding IDs, melting points, TLC characteristics, MS data, and yields are given in Table 2. The respective IR characteristics and ^1^H-NMR spectral data are presented in the Materials and Methods section.

### 2.2. Antioxidant Assays

#### 2.2.1. DPPH Radical Scavenging Assay

**Figure 3:**
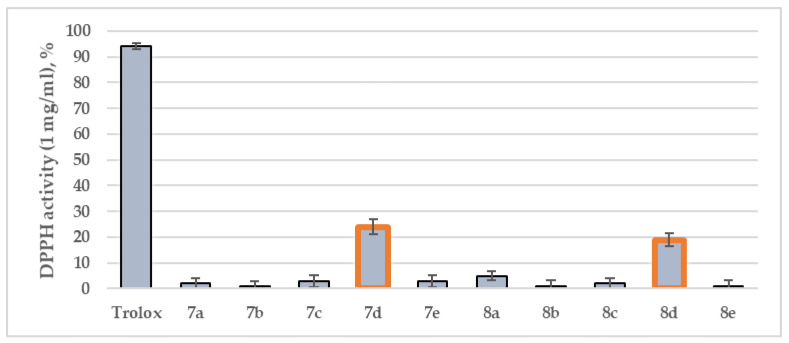
DPPH radical capacities of the newly synthesized compounds **7a**–**e** and **8a**–**e** at concentrations of 1 mg/mL. Trolox is used as an internal standard. Data are presented as means from three independent experiments. Standard deviation (SD) (n = 3).

The 1,1-diphenyl-2-picrylhydrazyl (DPPH) radical scavenging activity of the newly synthesized derivatives was determined at one concentration—1 mg/mL. Trolox was used as a standard. The obtained results are presented in Figure 3.

The highest DPPH scavenging activity was achieved by compound **7d** (24%). The *β*-alanine hydrazide–hydrazone condensed with the same aldehyde (**8d**) demonstrated similar radical scavenging activity (19%). The standard, Trolox, showed 94% DPPH activity. The rest of the newly synthesized molecules demonstrated weak to no antioxidant effects.

#### 2.2.2. ABTS Radical Scavenging Assay

**Figure 4:**
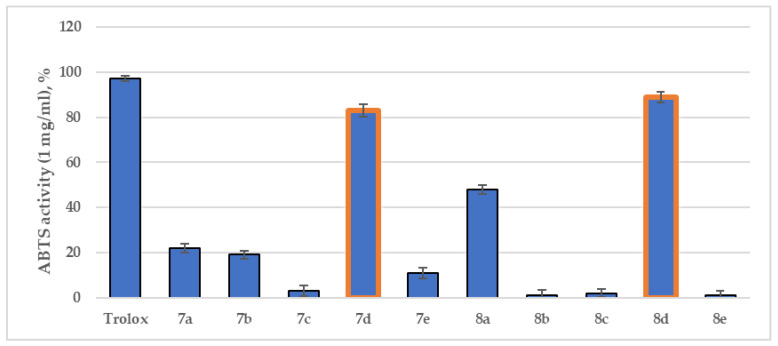
ABTS test of the newly synthesized compounds **7a**–**e** and **8a**–**e** at concentrations of 1 mg/mL. Trolox is used as an internal standard. Data are presented as means from three independent experiments. Standard deviation (SD) (n = 3).

During the 2,2′-azino-bis(3-ethylbenzothiazoline-6-sulphonic acid) (ABTS) assay, the discoloration of the initial color could be detected at 734 nm. The ABTS antioxidant assay of the title compounds is provided in Figure 4.

### 2.3. DFT Calculations

To rationalize the antioxidant assays and to determine the favored mechanism involved in free radical scavenging, density functional theory (DFT) calculations were carried out at the B3LYP/6-311++(d,p) level of theory.

#### 2.3.1. Optimized Geometries

**Figure 5:**
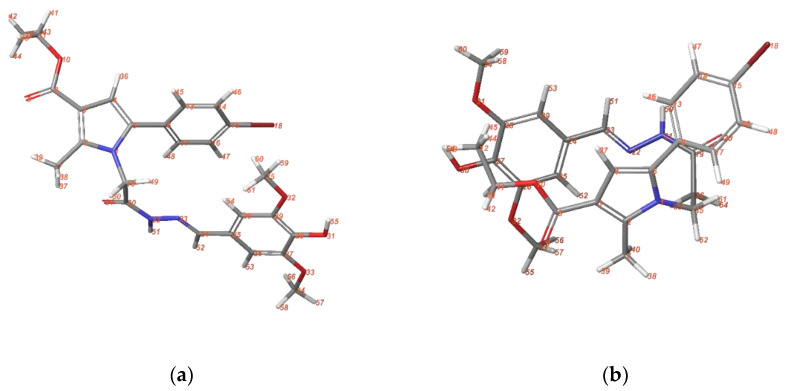
Final optimized geometries of the structures with 2500 iterations with OPLS4 force field. The best solutions for (**a**) **7d**; (**b**) **8d** are further optimized at B3LYP/6-311++(d,p).

The most prominent newly synthesized hydrazide–hydrazones (**7d** and **8d**) were selected for full optimization calculations. An initial conformational search has been performed by setting 2500 iterations with the OPLS4 force field. The best solutions were further optimized by full DFT geometry optimization at B3LYP/6-311++ (d,p) level of theory. The final geometries of **7d** and **8d** are visualized in Figure 5.

The overall minimized energies of **7d** and **8d** are −4197 and −4157 Hartree, respectively. It is important to note that *p*-bromophenyl moiety in structure **7d** was located in close proximity to the 3,5-dimethoxy-4-hydroxyphenyl fragment, while the same groups in **8d** were in different positions. In the latter case, the *p*-bromophenyl moiety faced away from the stable conformation. The most stable geometries have been selected as input geometries for further DFT studies.

#### 2.3.2. Analysis of Frontier Molecular Orbitals FMOs and Global Reactivity Descriptors

**Figure 6:**
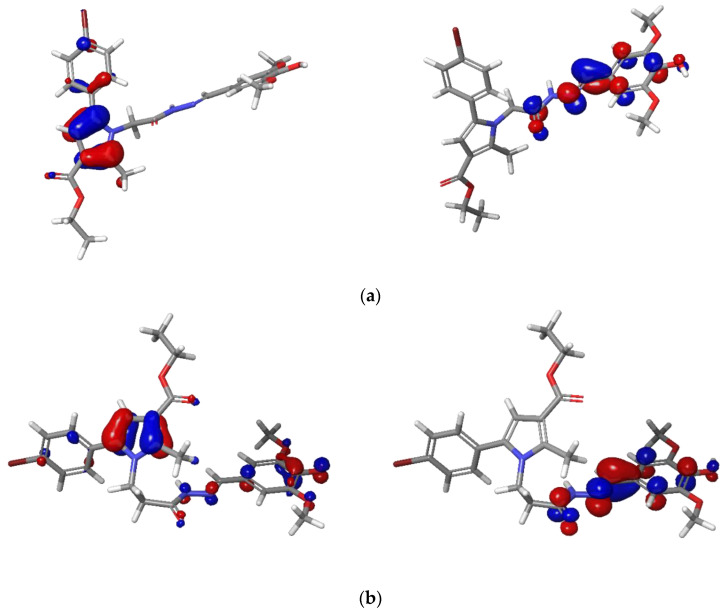
FMOs of **7d** and **8d**, calculated at DFT/B3LYP/6–311++(d,p): (**a**) HOMO and LUMO of **7d;** (**b**) HOMO and LUMO of **8d**. The HOMO-LUMO electronic densities are obtained from the optimized structures with DFT/B3LYP/6–311++(d,p) calculations. Blue color indicates a positive phase, and red indicates a negative phase.

A quantitative conceptual DFT analysis of the stability and reactivity of the investigated molecules (**7d** and **8d**) was carried out by calculations of the highest occupied molecular orbital (HOMO), the lowest unoccupied molecular orbital (LUMO), and subsequently, the global reactivity descriptors, such as ionization potential (IP), electron affinity (EA), molecular hardness and softness, electronegativity, and electrophilicity. The HOMO-LUMO electronic densities of **7d** and **8d** obtained from the optimized structures with DFT/B3LYP/6-311++(d,p) calculations are given in Figure 6. The blue color was used for a positive phase, and red was used for a negative phase.

It was found that the HOMO of both ligands is localized majorly on the pyrrole ring in **7d** and **8d**, while the LUMO is centered around the hydrazide–hydrazone moiety and the 3,5-dimethoxy-4-hydroxyphenyl fragment. The more energetically favorable conformation of **8d** (-4157 Hartree) led to a more compact structure and, therefore, the localization of HOMO is further spread to the hydrazide–hydrazone and the 3,5-dimethoxy-4-hydroxyphenyl moieties.

The energies (in atomic units) of the FMOs and the global reactivity descriptors (hardness (η), softness (S), electronegativity (χ), chemical potential (μ), and electrophilicity index (ω)) were calculated by applying the Koopman’s theorem for closed-shell compounds [16]. The data is reported in Table 3.

#### 2.3.3. Descriptors of the Antioxidant Properties

The calculated values for the dissociation of hydrogen-connected bonds and the IPs are provided in Table 4.

### 2.4. In Vitro Evaluations of the Cytotoxicity and Antioxidative Protective Activity on the SH-SY5Y Neuroblastoma Cell Line

#### 2.4.1. Effects of the Newly Synthesized Derivatives **7a**–**e** and **8a**–**e** on the SH-SY5Y Cell Viability

The effects of the newly synthesized N-pyrrolyl-hydrazone derivatives on the cellular viability of neuronal SH-SY5Y cells were evaluated, and the corresponding IC50 values were calculated. In the current study, the cells were seeded at density 2 × 10^4^ and were treated with the test compounds at concentrations 1–500 μM for 24 h. The calculated values of IC50 are presented in Table 5.

The results demonstrated that compounds **7c**, **7d**, and **8e** possess lackluster or low toxicity on human neuronal SH-SY5Y cells, with IC50 of >500 µM, 99.56 µM, and 91.07 µM, respectively.

#### 2.4.2. Effects of the Newly Synthesized Derivatives **7a**–**e** and **8a**–**e** in a Model of H_2_O_2_-Induced Oxidative Stress In Vitro

**Figure 7:**
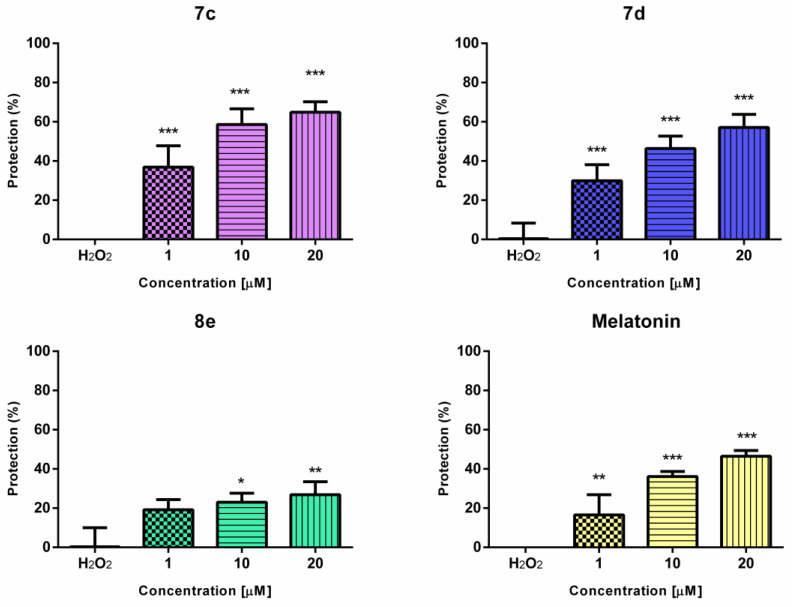
Protective effects of **7c**, **7d**, **8e**, and melatonin in a model of H_2_O_2_-induced oxidative damage in human neuroblastoma SH-SY5Y cells. Data are presented as means from three independent experiments ± SD (n = 8). * *p* < 0.05; ** *p* < 0.01; *** *p* < 0.001, vs. H_2_O_2_ group (one-way analysis of variance with Dunnet’s post hoc test).

The potential cell protective effects of **7c**, **7d**, and **8e** (the less cytotoxic compounds from the series) were evaluated against H_2_O_2_-induced oxidative stress in SH-SY5Y cells (Figure 7).

The cells were pre-incubated with the tested compounds at concentrations 1, 10, and 20 µM for 90 min. Melatonin was used as a reference compound because of its well-established neuroprotective effects in various in vitro and in vivo models [17]. After 90 min pre-incubation with the test substances, SH-SY5Y cells were exposed to H_2_O_2_ (1 mM, 15 min), as described in the Materials and Methods section.

The most potent protection was observed with compound **7c**, followed by **7d** and **8e**. Interestingly, both **7c** and **7d** showed better protection in all tested concentrations than the reference compound melatonin. It should be noted that compounds **7e** and **7d** showed statistically significant protection even at the lowest tested concentration (1 µM). Compound **8e** showed lower antioxidant protection.

## 3. Discussion

### 3.1. Synthesis

The selection of the applied synthetic path was based on literary data showing the relation between the acidity of the media and the condensation time. Initially, the synthesis was carried out in an ethanol medium without a catalyst, with reaction times of 50–60 min [18]. In an attempt to speed up the process, the same reactions were carried out in an ethanol medium with a catalyst of glacial acetic acid. Under these conditions, the hydrazones were obtained in lower yields of 26–64%, with no significant alteration of the reaction time [19]. Thus, Koopaei presents some data on the interaction of carbohydrazides with carbonyl compounds in an ethanol medium with hydrochloric acid as the catalyst at 100 °C. Applying these conditions, it took 20–30 min to complete the reaction [20,21]. The obtained products were isolated and recrystallized. These conditions did not change either the yield or the time of the condensation.

Thus, in the current work, we conducted the synthesis entirely in glacial acetic acid media. This allowed good yields and optimal time for obtaining pure products (Table 1).

### 3.2. Antioxidant Assays

Two of the most common assays for the assessment of antioxidant capacities of natural and/or synthetic molecules are the well-applied 2,2′-azino-bis(3-ethylbenzothiazoline-6-sulphonic acid) (ABTS) and 1,1-diphenyl-2-picrylhydrazyl (DPPH) assays. Both techniques are based on spectrophotometric measurement of the quenching of stable colored radicals (ABTS^•+^ or DPPH), which, in turn, allows evaluation of the radical scavenging ability of antioxidants even in complex mixtures [22].

#### 3.2.1. DPPH Radical Scavenging Assay

One of the most common assays for the evaluation of antioxidant effects is the DPPH test. Its frequent applications are mainly due to its relative inexpensiveness [23].

The scavenging activity in the 1,1-diphenyl-2-picrylhydrazyl (DPPH) assay is attributed to the hydrogen-donating ability of antioxidants with a following decrease in the absorbance at 517 nm. The most probable mechanisms of the DPPH·assay are based on hydrogen atom abstraction (HAT) [24], single electron transfer (SET) [25], or mixed mode [26]. The following reaction schemes are related to these mechanisms:

DPPH (violet at 515 nm) + ArOH → DPPHH (colorless) + ArO·HAT(1)

DPPH (violet at 515 nm) + ArOH → DPPH− (colorless) + ArO·+ SET(2)

as suggested in [27].

This allowed us to apply this method in order to evaluate the possible radical scavenging effects of the newly synthesized pyrrole-based hydrazones. The obtained results for the target hydrazones are presented in Figure 3.

#### 3.2.2. ABTS Radical Scavenging Assay

In the ABTS^•+^ radical scavenging assay (an electron-transfer-based assay), the 2,2′-azino-bis(3-ethylbenzothiazoline-6-sulfonate) radical cation (ABTS^•+^), which has a dark blue color, is reduced by an antioxidant into colorless ABTS, which can be measured spectrophotometrically at 734 nm [28].

A literary-based comparative analysis suggested two possible mechanisms of radical scavenging reactions related to the ABTS assay: formation of coupling adducts with ABTS^•+^—reaction considered as more characteristic for antioxidants of phenolic nature and oxidation without coupling. In addition, the data suggested the possibility for further oxidative degradation of the obtained coupling adducts, leading to hydrazindyilidene-like and/or imine-like adducts with 3-ethyl-2-oxo-1,3-benzothiazoline-6-sulfonate and 3-ethyl-2-imino-1,3-benzothiazoline-6-sulfonate as marker compounds, respectively [29].

Considering the presence of a phenyl residue in the structure of the new pyrrole-based hydrazones, it was of interest to compare the possible radical scavenging activity of the target compounds when determined through both the most common in vitro antioxidant assays (Figure 3 and Figure 4). The results from our evaluations demonstrated that when compared to the DPPH test, the ABTS assay revealed significantly better antioxidant capacities for the title compounds. The most prominent antioxidant was **8d**, with 89% scavenging activity, while **7d** showed 83%. Moreover, the hydrazide–hydrazones condensed with 4-dimethylamino benzaldehyde and **7a** and **8a** demonstrated moderate ABTS scavenging capacities of 22% and 48%, respectively. The drastic differences between the two assays could be related to the ability of the ABTS test to examine both hydrophobic and hydrophilic compounds, as well as to test bulky structures [30]. However, the application of the DPPH assay is still used by numerous research groups to produce significant and comparable antioxidant results [31].

In general, the best radical scavenging effect of compounds **7d** and **8d** is quite expected due to the presence of a free *p*-hydroxyl group in the phenyl part of the structure. The appearance of weak radical scavenging properties in the ABTS assay for compounds **7a** and **8a** is probably due to the free electron pair in the N-atom at the *p*-dimethylamino fragment of the phenyl residue. In addition, a negligible effect is observed for **7b**, containing an electron-withdrawing halogen Cl. The presence of *o*-methoxy groups in the phenyl residue is related to the lack of radical scavenging effects in **7e** and **8e**, probably due to the less likely possibility of these groups creating intermolecular hydrogen bonds.

It should also be mentioned that, in general, the shorter series **7a**–**e** is related to better radical scavenging activity in comparison to the one methylene longer series **8a**–**e**. This may be due to additional conformational changes leading to the hindrance of the possibility of hydrogen atom transfer and/or electron transfer ability of the active functional groups.

### 3.3. DFT Calculations

Several mechanisms for free-radical scavenging action of phenyl-containing molecules are proposed, where it is reported that phenolic O–H bond dissociation enthalpy (BDE), adiabatic ionization potential (IP), proton dissociation enthalpy (PDE), proton affinity (PA), and electron-transfer enthalpy (ETE) are important factors used to evaluate the preferred free-radical scavenging pathways of such structures thermodynamically [32]. In an attempt to clarify these mechanisms, some theoretical approaches are applied, with density functional theory (DFT) being most helpful in the calculation of these physicochemical descriptors [33].

The energies of the frontier molecular orbitals and the global reactivity descriptors were calculated with the B3LYP hybrid functional considering recent findings [32]. The corresponding HOMO and LUMO values provide information about the energy distribution and the energetic behavior of the **7d** and **8d**. The comparatively high negative value of HOMO emphasizes the high ability of both compounds to donate electrons to empty molecular orbital energy. Moreover, the negative magnitude of E_HOMO_ and E_LUMO_ establishes the stability of the compounds [34]. The energy gap of the FMOs (E_HOMO_–E_LUMO_) corresponds to the chemical reactivity and the kinetic stability. The calculated frontier molecular energy gaps are 0.1449 a.u and 0.1506 a.u for **7d** and **8d**, respectively. The large gap accounts for hard, unreactive, and less polarizable molecules.

The value of chemical softness (S) and hardness (η) identifies whether the compounds are reactive and can donate electrons. These descriptors are directly correlated with molecular stability and chemical reactivity. When compared, **7d** possesses slightly lower chemical hardness, which corresponds to lower stability. The calculated value of softness revealed that **8d** is more favorable in the charge–transfer mechanism. The negative value of the chemical potential of both ligands (−0.1288 and −0.1223) establishes good stabilities and resistance to sudden decomposition into their elements. The calculated electrophilicity index values (0.1145 and 0.0992 Ha), which are related to chemical potential and hardness, are indicative of the nucleophilicity power [35].

The DFT studies have indicated that the best radical scavenging ligands (**7d** and **8d**) in the newly synthesized molecules are stable, which do not decompose into elements, are less polarizable, and with hard nature. The energy of HOMO revealed that both compounds possess good electron donation capacities.

To examine the most probable mechanism of antioxidant activity of **7d** and **8d**, two processes were observed: hydrogen atom transfer (HAT) and single electron transfer (SET).

The bond dissociation energies (BDE) characterize the viability of hydrogen donation to the free radical. It is considered one of the most important descriptors in the process of determining the antioxidant effects [36]. Thus, the BDE was used as the best reliable thermodynamic parameter describing the hydrogen atom transfer (HAT) mechanism. Because this route involves the H atom transferring from a hydroxyl group of an antioxidant compound to the free radical, the weakest O–H bond with the lowest BDE is expected to be abstracted easier, revealing its higher antiradical (antioxidant) activity. Therefore, a computational calculation of the BDEs of available −OH groups in **7d** and **8d** will benefit in characterizing the theoretical antioxidant mechanism of the compounds. The activity of the −OH groups in both active ligands and the formation of stable radicals was conducted by calculating the dissociation energies.

In addition, apart from the HAT mechanism, another possible pathway for antioxidant molecules is single electron transfer followed by proton transfer (SET-PT). In this route, an electron is transferred from the antioxidant to the free radical, leading to radical cation formation, which subsequently deprotonates. Hence, adiabatic ionization proton (IP) and PDE are the most important parameters in describing the feasibility of the mechanism. In general, lower IPs are more subject to ionization and easier in electron-transfer rate between free radicals and antioxidants [33]. SET is related to the conceptual DFT parameters, mainly the IP energies [37]. This order reveals that compounds **7d** and **8d** are the most active antioxidants, with all the other calculated IPs being larger than the reference compound Trolox, suggesting their lower activity than Trolox.

The computationally calculated BDEs values of all possible dissociation sites in **7d** and **8d** revealed that radical formation occurs at O_31_, followed by C_7_ and N_21_. A formation of possible intramolecular stabilization forces could conclude the result of strong N-H bond dissociation in the hydrazide moiety. The O−H bond is the weakest one in both **7d** and **8d**, which describes the potential HAT mechanism in the exerted antioxidant activity. The IPs energies are responsible for the electron donation abilities of the title ligands. The former values of both compounds were higher when compared to the BDEs. Moreover, the relatively low IP energies of the pyrrole-based ligands [38] imply the substantial contribution of the SET mechanism in the antioxidant capacities. Overall, the DFT calculations demonstrated that **7d** and **8d** possess similar values of BDEs and IPs, which provides a good theoretical explanation for the experimental, radical scavenging assays.

### 3.4. In Vitro Cytotoxicity and Antioxidative Protective Activity on SH-SY5Y Cells

#### 3.4.1. Effects of the Newly Synthesized Derivatives **7a**–**e** and **8a**–**e** on SH-SY5Y Cell Viability

Toxicity evaluation of newly synthetized substances in different in vitro models is an important issue in the drug development process.

In the current study, we selected the human neuroblastoma cell line SH-SY5Y as an appropriate model for in vitro neurotoxicity evaluation due to its frequent use in experimental neuroscience [39]. This cell line is also an appropriate model for mimicking various neurodegenerative disorders since its cells are able to convert to numerous types of functional neurons by the introduction of specific compounds. In addition, the SH-SY5Y cells possess tyrosine hydroxylase and dopamine-*β*-hydroxylase activities because of their sympathetic adrenergic ganglia origin [39], thus making it appropriate for evaluation of dopamine and serotonin modulations in neurodegeneration.

We aimed to evaluate the effects of the newly synthesized N-pyrrolyl hydrazone derivatives **7a**–**e** and **8a**–**e** on the cellular viability of the neuronal SH-SY5Y cells and calculated the corresponding IC50 values (Table 5). The results indicated three compounds with low toxicity; among them, compound **7c** may be considered non-toxic with effects comparable to the ones of melatonin. Interestingly, the results showed that the prolongation of the methylene bridge between the central pyrrole ring and the azomethine fragment of the structure with one carbon atom is related to a slight increase in cellular toxicity. The obtained IC50 values showed that the overall cytotoxicity of compounds from series **8a**–**e** is slightly higher compared to series **7a**–**e**. Particularly, the toxicity of derivative **8d** is 1.7 times higher than the one of its monomethyl analogue **7d**. Based on their promising safety profile, the compounds **7c**, **7d**, and **8e** were chosen for the further antioxidative protection assay in vitro.

#### 3.4.2. Effects of the Newly Synthesized Derivatives **7a**–**e** and **8a**–**e** in a Model of H_2_O_2_-Induced Oxidative Stress In Vitro

Oxidative stress plays a key role in the damage to the central nervous system and in the pathogenesis of neurodegenerative diseases [40]. When an imbalance between the production of free radicals and detoxification occurs, the high production of ROS can overcome the body’s antioxidant defenses, leading to harmful conditions such as oxidative stress and damage to cellular structures and functions.

In addition, free radical overproduction is often considered to be involved in the acute damage of the central nervous system and is strictly related to the development of neurodegenerative diseases. There are a number of facts that determine the free radical overproduction as a direct cause of immature cultured cortical neurons apoptosis [41], induction of DNA damage [42], and necrotic cell death [43], among others [44].

H_2_O_2_ is one of the most important ROS generated through oxidative stress. It causes oxidative damage in different cell structures such as nucleic acids, proteins, and membrane lipids; therefore, it is commonly used as an in vitro model for inducing oxidative cell damage [45]. The main mechanism of induction of oxidative stress relates to the production of reactive hydroxyl radicals and the formation of Fenton’s reaction, which interacts directly with proteins, lipids, and DNA.

The less cytotoxic compounds **7c**, **7d**, and **8e** were tested for antioxidant protection on the H_2_O_2_-induced oxidative stress model in neuroblastoma SH-SY5Y cells. As expected, H_2_O_2_-treatment (1 mM, 15 min) induced a significant decrease in SH-SY5Y cell viability. Our results demonstrated that the pre-treatment with compounds **7c**, **7d**, and **8e** significantly increased the SH-SY5Y cell survival compared to H_2_O_2-_ treatment. Both melatonin and all three test compounds show dose-dependent protective effects. Compared to the reference compound melatonin, at all the applied concentrations between 1 and 20 µM, compounds **7c** and **7d** both exert stronger protective effects, and **8e**’s effect is lower than melatonin’s. Noteworthy, despite the stronger radical scavenging activity of **7d** compared to **7c**, shown by both the DPPH and ABTS assay, both compounds cause similar in vitro protective effects on peroxide-damaged SH-SY5Y cells, which may indicate that in the cells, the compounds have pleiotropic modes of antioxidant action.

The overall results from the performed in vitro assessment of the radical scavenging activity and antioxidative protective effects on neuronal SH-SY5Y cells of the newly synthesized N-pyrrolyl hydrazones determined the ethyl 5-(4-bromophenyl)-1-(2-(2-(4-hydroxy-3,5-dimethoxybenzylidene)hydrazine-yl)-2-oxoethyl)-2-methyl-1H-pyrrole-3-carboxylate (**7d**) as the most perspective structure with lowest cytotoxicity, highest radical scavenging activity and best antioxidative protection on H_2_O_2_-induced oxidative stress model.

### 3.5. Limitations and Implications Section

Some limitations of the applied assays are related to the specificity of the radical scavenging evaluations used, such as the fact that DPPH and ABTS are not physiological radicals; thus, they are not similar to peroxyl-radicals or other oxygen-based radicals. This makes the test an indirect method based on the reduction of persistent radicals. Thus, some discrepancies may be observed since light, oxygen, and pH have an influence on the final absorbance. In this relation, we plan to add some additional cellular evaluations on the effect of the newly obtained substances on cell-defined oxidative mechanisms, which will add to the performed cell protective experiments on H_2_O_2_-induced oxidative stress applied here. These evaluations include 6-hydroxydopamine (6-OHDA)-, tert-butyl hydroperoxide (tBuOOH)-, and Fe^2+^ascorbate-induced oxidative stress assays on cellular and sub-cellular levels, which will be published in a following study.

## 4. Materials and Methods

### 4.1. Materials

The purity of the obtained compounds and the progress of the reactions were controlled by thin layer chromatography (TLC) on TLC-CardsSilicagel 60 F254, 1.05554, Merck, Darmstadt, Germany, using CHCl_3_/CH_3_CH_2_OH as a mobile phase. Melting points were determined in open capillary tubes on an IA 9200 ELECTROTHERMAL apparatus, Southend-on-Sea, England. All chemical names are given according to IUPAC by the program ChemBioDraw Ultra software, Version 11.0, CambridgeSoft. The IR spectra were performed in the range 400–4000 cm^−1^ on a NicoletiS10 FT-IR spectrophotometer, using ATR technique with Smart iTR adapter. ^1^H-NMR spectra were recorded on a Bruker-Spectrospin WM250 MHz, Faelanden, Switzerland, operating at 250 MHz, as *δ* (ppm) relative to TMS as internal standard. Mass spectra were registered on a 6410 Agilent LCMS triple quadrupole mass spectrometer (LCMS) with an electrospray ionization (ESI) interface. Elemental analyses were performed on a Euro EA 3000-Single, EUROVECTOR SpAanalyser. All chemicals and reagents used as starting materials were purchased from Merck (Darmstadt, Germany).

The necessary for the pharmacological evaluations RPMI cell culture medium, heat-inactivated fetal bovine serum (FBS), L-glutamine, (3-(4,5-dimethylthiazol-2-yl)-2,5- diphenyltetrazolium bromide (MTT), hydrogen peroxide (H_2_O_2_), and dimethyl sulfoxide (DMSO) were acquired from Sigma-Aldrich (Merck KGaA, Darmstadt, Germany).

### 4.2. General Synthesis of the New Compounds

The corresponding N-pyrrolyl-carbohydrazide **7** or **8** and any of the carbonyl compounds **a**, **b**, **c**, **d**, or **e** (Figure 2) were incubated in a glacial acetic acid in a round bottom flask of 50 mL and stirred at 100 °C to complete the reaction under TLC-control. The obtained products were isolated, washed with diethyl ether, and recrystallized, where necessary, by ethanol.

#### 4.2.1. (E)-ethyl 5-(4-bromophenyl)-1-(2-(2-(4-(dimethylamino)benzylidene)hydrazinyl)-2-oxoethyl)-2-methyl-1H-pyrrole-3-carboxylate (**7a**)

Yield: 84% as an orange powder; m.p. 211.4–213.6; IR (cm^−1^): 3245 (NH), 2976 (CH_3_ and CH_2_), 1701 (COOC_2_H_5_), 1666 (Amide I), 1533 (Amide II), 1232 (C-O), 1073 (C-N), 810 (*p*-substituted C_6_H_4_), 552 (C-Br); ^1^HNMR (δH, 250 MHz, CDCl_3_): 1.99 [s, 3H, CH_2_CH_3_], 2.54 [s, 3H, CH_3_(2)], 3.12–3.15 [m, 6H, N(CH_3_)2], 4.20–4.24 [m, 2H, CH_2_CH_3_], 4.82 [s, 2H, CH_2_CO], 6.53 [s, 1H, H(4)], 7.19 [s, 2H, H(3″), H(5″)], 7.21 [s, 2H, H(2″), H(6″)], 7.68–7.69 [d, 2H, H(3′), H(5′)], 7.88–7.90 [d, 2H, H(2′), H(6′)], 9.99 [s, 2H, NH-N=CH]; LC-MS (ESI): Calc. for C_25_H_28_O_3_N_4_Br [M+H]^+^: 511.1339; Found: 511.1339.; Anal. Calc. for C_25_H_27_BrN_4_O_3_: C, 58.71; H, 5.32. Found: C, 58.68; H, 5.34.

#### 4.2.2. (E)-ethyl 5-(4-bromophenyl)-1-(2-(2-(4-chlorobenzylidene)hydrazinyl)-2-oxoethyl)-2-methyl-1h-pyrrole-3-carboxylate (**7b**)

Yield: 78% as a white powder; m.p. 229.4–231.2; IR (cm^−1^): 3179 (NH), 2998 (CH_3_ and CH_2_), 1697 (COOC_2_H_5_), 1667 (Amide I), 1570 (Amide II), 1240 (C-O), 816 (*p*-substituted C_6_H_4_), 772 (C-Cl), 557 (C-Br); ^1^HNMR (δH, 250 MHz, DMSO-d_6_): 1.25–1.29 [m, 3H, CH_2_CH_3_], 2.44–2.48 [m, 3H, CH_3_(2)], 4.14–4.23 [m, 2H, CH_2_CH_3_], 5.10 [s, 2H, CH_2_CO], 6.49 [s, 1H, H(4)], 7.23–7.27 [m, 1H, H(3″)], 7.31–7.36 [m, 1H, H(5″)], 7.46–7.47 [d, 1H, H(6′)], 7.50–7.51 [d, 1H, H(2′)], 7.58 [s, 1H, H2″)], 7.59 [s, 1H, H(6″)], 7.68 [ s, 1H, H(3′)], 7.71 [s, 1H, H(5′)], 8.0 [s, 1H, CH=N], 11.81 [s, 1H, CONH]; LC-MS (ESI): Calc. for C_23_H_22_O_3_N_3_BrCl [M+H]^+^: 502.0527; Found: 502.0528.; Anal. Calc. for C_23_H_21_BrClN_3_O_3_: C, 54.94; H, 4.21. Found: C, 54.84; H, 4.29.

#### 4.2.3. (E)-ethyl 5-(4-bromophenyl)-2-methyl-1-(2-(2-(4-nitrobenzylidene)hydrazinyl)-2-oxo-ethyl)-1H-pyrrole-3-carboxylate (**7c**)

Yield: 76% as a white powder; m.p. 245.9–247.2; IR (cm^−1^): 3201 (NH), 3064 (CH_3_ and CH_2_), 1678 (COOC_2_H_5_), 1592 (Amide I), 1558 (Amide II), 1348 (NO_2_), 1240 (C-O), 1205 (C-N), 814 (*p*-substituted C_6_H_4_), 556 (C-Br); ^1^HNMR (δH, 250 MHz, CDCl_3_): 1.35–1.37 [m, 3H, CH_2_CH_3_], 2.58 [s, 3H, CH_3_(2)], 4.92 [s, 2H, CH_2_CH_3_], 5.05 [s, 2H, CH_2_CO], 6.65 [s, 1H, H(4)], 7.58 [s, 1H, H(5′)], 7.59 [s, 1H, H(3′)], 7.78–7.93 [m, 2H, H(2′), H(6′)], 8.28 [s, 1H, H(2″)], 8.30 [s, 1H, H(6″)], 8.40 [s, 1H, H(3″)], 8.41 [s, 1H, H(5″)], 10.18 [s, 2H, NH-N=CH]; LC-MS (ESI): Calc. for C_23_H_22_O_5_N_4_Br [M+H]^+^: 513.0768; Found: 513.0766; Anal. Calc. for C_23_H_21_BrN_4_O_5_: C, 53.81; H, 4.12. Found: C, 53.78; H, 4.10.

#### 4.2.4. (E)-ethyl 5-(4-bromophenyl)-1-(2-(2-(4-hydroxy-3,5-dimethoxybenzylidene)hydrazine-yl)-2-oxoethyl)-2-methyl-1H-pyrrole-3-carboxylate (**7d**)

Yield: 68% as a white powder; m.p. 191.9- 194.4; IR (cm^−1^): 3424 (OH), 3208 (NH), 3067 (CH_3_ and CH_2_), 1694 (COOC_2_H_5_), 1673 (Amide I), 1570 (Amide II), 1236 (C-O), 814 (*p*-substituted C_6_H_4_), 554 (C-Br); ^1^HNMR (δH, 250 MHz, DMSO-d_6_): 1.29 [t, 3H, CH_2_CH_3_], 2.47 [s, 3H, CH_3_(2)], 3.77 [s, 6H, OCH_3_(3″), OCH_3_(5″)], 4.15–4.21 [m, 2H, CH_2_CH_3_], 5.05 [s, 1H, OH], 5.09 [s, 2H, CH_2_CO], 6.50 [s, 1H, H(4)], 6.93 [s, 1H, H(6″)], 6.98 [s, 1H, H(2″)], 7.28–7.38 [m, 2H, H(3′), H(5′)], 7.59–7.63 [m, 2H, H(2′), H(6′)], 7.89 [s, 1H, CH=N], 11.67 [s, 1H,CONH]; LC-MS (ESI): Calc. for C_25_H_27_O_6_N_3_Br [M+H]^+^: 544.1078; Found: 544.1078; Anal. Calc. for C_25_H_26_BrN_3_O_6_: C, 55.16; H, 4.81. Found: C, 55.15; H, 4.85.

#### 4.2.5. (E)-ethyl 5-(4-bromophenyl)-1-(2-(2-(2,4-dimethoxybenzylidene)hydrazinyl)-2-oxo-ethyl)-2-methyl-1H-pyrrole-3-carboxylate (**7e**)

Yield: 72% as a white powder; m.p. 206.0–207.6; IR (cm^−1^): 3234 (NH), 2984 (CH_3_ and CH_2_), 1698 (COOC_2_H_5_), 1668 (Amide I), 1520 (Amide II), 1240 (C-O), 822 (*p*-substituted C_6_H_4_), 554 (C-Br); ^1^HNMR (δH, 250 MHz, CDCl_3_): 1.36 [t, 3H, CH_2_CH_3_], 2.54 [s, 3H, CH_3_(2)], 3.93 [s, 3H, OCH_3_(2″)], 3.94 [s, 3H, OCH_3_(4″)], 4.29–4.31 [m, 2H, CH_2_CH_3_], 4.92 [s, 2H, CH_2_CO], 6.64 [s, 1H, H(4)], 6.47 [s, 1H, H(3″)], 6.48 [s, 1H, H(5″)], 7.27 [s, 1H, H(6″)], 7.56 [s, 1H, H(2′)], 7.57 [s, 1H, H(6′)], 7.82 [s, 1H, H(3′)], 7.85 [s, 1H, H(5′)], 10.31 [s, 1H, CH=N], 10.32 [s, 1H, CONH]; LC-MS (ESI): Calc. for C_25_H_27_O_5_N_3_Br [M+H]^+^: 528.1129; Found: 528.1132; Anal. Calc. for C_25_H_26_BrN_3_O_5_: C, 56.83; H, 4.96. Found: C, 56.84; H, 4.98.

#### 4.2.6. (E)-ethyl 5-(4-bromophenyl)-1-(3-(2-(4-(dimethylamino)benzylidene)hydrazinyl)-3-oxopropyl)-2-methyl-1H-pyrrole-3-carboxylate (**8a**)

Yield: 86% as a white powder; m.p. 212.0–213.3; IR (cm^−1^): 3266 (NH), 2907 (CH_3_ and CH_2_), 1693 (COOC_2_H_5_), 1668 (Amide I), 1570 (Amide II), 1265 (C-N), 1249 (C-O), 831 (*p*-substituted C_6_H_4_), 554 (C-Br); ^1^HNMR (δH, 250 MHz, CDCl_3_): 1.27 [s, 3H, CH_2_CH_3_], 2.54 [s, 3H, CH_3_(2)], 2.59–2.62 [m, 2H, CH_2_CH_2_CO], 3.12 [s, 3H, N(CH_3_)], 3.14 [s, 3H, N(CH_3_)], 4.17–4.19 [m, 2H, CH_2_CH_2_CO], 4.27–4.28 [m, 2H, CH_2_CH_3_], 6.49 [s, 1H, H(4)], 7.18–7.19 [m, 2H, H(3″), H(5″)], 7.45–7.49 [m, 2H, H(2″), H(6″)], 7.67–7.69 [d, 2H, H(3′), H(5′)], 7.95–7.96 [d, 2H, H(2′), H(6′)], 8.60 [s, 1H, CH=N], 9.97 [s, 1H, CONH]; LC-MS (ESI): Calc. for C_26_H_30_O_3_N_4_Br [M+H]^+^: 525.1496; Found: 525.1504; Anal. Calc. for C_26_H_29_BrN_4_O_3_: C, 59.43; H, 5.56. Found: C, 59.23; H, 5.66.

#### 4.2.7. (E)-ethyl 5-(4-bromophenyl)-1-(3-(2-(4-chlorobenzylidene)hydrazinyl)-3-oxopropyl)-2-methyl-1H-pyrrole-3-carboxylate (**8b**)

Yield: 74% as a white powder; m.p. 181.4–184.6; IR (cm^−1^): 3282 (NH), 2931 (CH_3_ and CH_2_), 1698 (COOC_2_H_5_), 1668 (Amide I), 1571 (Amide II), 1252 (C-O), 813 (*p*-substituted C_6_H_4_), 771 (C-Cl), 560 (C-Br); ^1^HNMR (δH, 250 MHz, DMSO-d_6_): 1.23–1.27 [m, 3H, CH_2_CH_3_], 2.61 [s, 3H, CH_3_(2)], 2.83 [t, 2H, CH_2_CH_2_CO], 4.13–4.16 [q, 2H, CH_2_CH_2_CO], 4.24–4.33 [q, 2H, CH_2_CH_3_], 6.41 [s, 1H, H(4)], 7.35–7.37 [d, 2H, H(3″), H(5″)], 7.51–7.53 [d, 2H, H(2″), H(6″)], 7.56–7.60 [m, 2H, H(2′), H(6′)], 7.61–7.63 [d, 1H, H(3′)], 7.69–7.71 [d, 1H, H(5′)], 7.86 [s, 1H, CH=N], 11.38 [s, 1H, CONH]; LC-MS (ESI): Calc. for C_24_H_24_O_3_N_3_BrCl [M+H]^+^: 516.0684; Found: 516.0684; Anal. Calc. for C_24_H_23_BrClN_3_O_3_: C, 55.78; H, 4.49. Found: C, 55.76; H, 4.48.

#### 4.2.8. (E)-ethyl 5-(4-bromophenyl)-2-methyl-1-(3-(2-(4-nitrobenzylidene)hydrazinyl)-3-oxopropyl)-1H-pyrrole-3-carboxylate (**8c**)

Yield: 82% as a yellow powder; m.p. 214.4–217.1; IR (cm^−1^): 3294 (NH), 2929 (CH_3_ and CH_2_), 1689 (COOC_2_H_5_), 1658 (Amide I), 1572 (Amide II), 1512 (NO_2_), 1340 (C-N), 1249 (C-O), 814 (*p*-substituted C_6_H_4_), 558 (C-Br); ^1^HNMR (δH, 250 MHz, CDCl_3_): 1.26–1.29 [m, 3H, CH_2_CH_3_], 2.55 [s, 3H, CH_3_(2)], 2.61 [t, 2H, CH_2_CH_2_CO], 4.18–4.20 [m, 2H, CH_2_CH_2_CO], 4.21–4.25 [m, 2H, CH_2_CH_3_], 6.49 [s, 1H, H(4)], 7.17 [s, 1H, H(3’)], 7.18 [s, 1H, H(5’)], 7.48 [s, 1H, H(6’)], 7.49 [s, 1H, H(2’)], 8.00 [s, 1H, H(2″)], 8.02 [s, 1H, H(6″)], 8.34 [s, 2H, H(3″), H(5″)], 10.09 [s, 2H, NH-N=CH]; LC-MS (ESI): Calc. for C_24_H_24_O_5_N_4_Br [M+H]^+^: 527.0925; Found: 527.0927; Anal. Calc. for C_24_H_23_BrN_4_O_5_: C, 54.66; H, 4.40. Found: C, 54.64; H, 4.39.

#### 4.2.9. (E)-ethyl 5-(4-bromophenyl)-1-(3-(2-(4-hydroxy-3,5-dimethoxybenzylidene) hydra zine-yl)-3-oxopropyl)-2-methyl-1H-pyrrole-3-carboxylate (**8d**)

Yield: 64% as a white powder; m.p. 196.6–197.6; IR (cm^−1^): 3422 (OH), 3278 (NH), 2971 (CH_3_ and CH_2_), 1693 (COOC_2_H_5_), 1666 (Amide I), 1574 (Amide II), 1250 (C-O), 814 (*p*-substituted C_6_H_4_), 551 (C-Br); ^1^HNMR (δH, 250 MHz, DMSO-d_6_): 1.22–1.27 [m, 3H, CH_2_CH_3_], 2.61 [t, 2H, CH_2_CH_2_CO], 2.64 [s, 3H, CH_3_(2)], 3.78 [s, 6H, OCH_3_(3″), OCH3(5″)], 4.12–4.16 [m, 2H, CH_2_CH_2_CO], 4.20–4.26 [m, 2H, CH_2_CH_3_], 6.41 [s, 1H, H(4)], 6.80 [s, 1H, OH], 7.36–7.38 [m, 2H, H(2″), H(6″)], 7.54–7.56 [m, 2H, H(3′), H(5′)], 7.62–7.64 [m, 2H, H(2′), H(6′)], 7.78 [s, 1H, CH=N], 11.20 [s, 1H,CONH]; LC-MS (ESI): Calc. for C_26_H_29_O_6_N_3_Br [M+H]^+^: 558.1234; Found: 558.1243; Anal. Calc. for C_26_H_28_BrN_3_O_6_: C, 55.92; H, 5.05. Found: C, 55.98; H, 5.02.

#### 4.2.10. (E)-ethyl 5-(4-bromophenyl)-1-(3-(2-(2,4-dimethoxybenzylidene)hydrazinyl)-3-oxopropyl)-2-methyl-1H-pyrrole-3-carboxylate (**8e**)

Yield: 74% as a white powder; m.p. 170.0–171.9; IR (cm^−1^): 3298 (NH), 2968 (CH_3_ and CH_2_), 1692 (COOC_2_H_5_), 1673 (Amide I), 1571 (Amide II), 1253 (C-O), 813 (*p*-substituted C_6_H_4_), 549 (C-Br); ^1^HNMR (δH, 250 MHz, CDCl_3_): 1.24–1.28 [m, 3H, CH_2_CH_3_], 2.54 [s, 3H, CH_3_(2)], 2.58–2.63 [m, 2H, CH_2_CH_2_CO], 3.18 [s, 3H, OCH_3_(4″)], 3.83 [s, 3H, OCH_3_(2″)], 4.18–4.20 [m, 2H, CH_2_CH_2_CO], 4.21–4.23 [m, 2H, CH_2_CH_3_], 6.37–6.68 [d, 1H, H(3″)], 6.47–6.48 [m, 1H, H(5″)], 6.49 [s, 1H, H(4)], 7.14 [s, 1H, H(3′)], 7.18 [s, 1H, H(5′)], 7.48 [s, 1H, H(6′)], 7.49 [s, 1H, H(2′)], 7.73–7.76 [d, 1H, H(6″)], 8.34 [s, 1H, CH=N], 10.22 [s, 1H, CONH]; LC-MS (ESI): Calc. for C_26_H_29_O_5_N_3_Br [M+H]^+^: 542.1285; Found: 542.1293; Anal. Calc. for C_26_H_28_BrN_3_O_5_: C, 57.57; H, 5.20. Found: C, 57.55; H, 5.22.

### 4.3. Antioxidant Activity Evaluation

#### 4.3.1. DPPH Radical Scavenging Assay

The scavenging assay of the title compounds against DPPH radical was carried out by the widely employed protocol of Brand-Williams et al. [46]. Briefly, a single concentration of 1 mg/mL for each synthesized compound in methanol was obtained. Subsequent addition of 1 mL of the methanol solution of DPPH (1 mmol/L) was performed. The reaction mixtures were incubated in the dark for 30 min. The absorbance was measured at 517 nm. Three measurements were carried out for each sample; 6-Hydroxy-2,5,7,8- tetramethylchroman-2-carboxylic acid (Trolox) was applied as a standard. The percentage inhibition of the tested samples was calculated by the following Formula (3):

DPPH_scavenging activity_ = Abs_control_ − Abs_sample_/Abs_control_ × 100% (3)

where Abs_control_ is the absorbance of the DPPH radical in methanol, and Abs_sample_ is the absorbance of the DPPH radical solution mixed with the sample.

#### 4.3.2. ABTS Radical Scavenging Assay

The ABTS radical tests were measured according to a modified method of Arnao et al. [47]. The test solutions were dissolved in methanol (1 mg/mL) at ambient temperature. The radical cation of ABTS (ABTS^+•^) was created by mixing 7 mmol/L solution of ABTS and 2.4 mmol/L solution of potassium persulphate, which were set to react for 14 h in the dark at room temperature. The working solutions consisted of 2 mL of the stock solution diluted in 50 mL of methanol with an absorbance of 0.294 ± 0.05 units at 734 nm. Then, 1 mL of the ABTS working solution was allowed to react with the title compounds for 10 min, with a subsequent absorbance determination. The inhibition percentages were evaluated by applying the same formula as the DPPH assay (4).

ABTS_scavenging activity_ = Abs_control_ − Abs_sample_/Abs_control_ × 100% (4)

where Abs_control_ is the absorbance of the ABTS radical in methanol, and Abs_sample_ is the absorbance of the ABTS radical solution mixed with the sample.

### 4.4. DFT Theoretical Calculations

All of the theoretical computations were carried out by applying Jaguar [48]. The initial geometries for the DFT calculations were achieved after a conformational search with 2500 iterations and OPLS4 force field. Subsequently, full geometry optimizations of the best conformations of compounds **7d** and **8d** with Becke’s three-parameter hybrid exchange–correlation functional (B3LYP) and 6-311++G (d,p) basis set in the gas phase were performed. It was found that out of 11 functions and 14 basis sets, the most optimal combination in terms of both accuracy and resource usage for bond dissociation energies calculations is M06-2X/6-311G(d,p) [49]. The frontier molecular orbitals and the global reactivity descriptors were calculated at the same level of theory. The bond dissociation energies were obtained with M06–2X and 6–311G basis set considering a recent report [49].

### 4.5. In Vitro Pharmacological Evaluations

#### 4.5.1. Cell Line

Human neuroblastoma cell line SH-SY5Y was purchased from European Collection of Cell Cultures (ECACC, Salisbury, UK). SH-SY5Y cells were cultivated in an RPMI 1640 medium supplemented with 10% heat-inactivated FBS, 2 mM L-glutamine, and 1% antibiotics (penicillin/streptomycin). The cell line was incubated at 37 °C with 5% of CO_2,_ and the culture’s medium was replaced with a time interval of 2–3 days.

#### 4.5.2. Cell Viability Assay

The SH-SY5Y cells were plated for 24 h at 37 °C on 96-well plates at a density of 2 × 10^4^ cells per well to confluence. After 24 h, the cells were treated with the compounds (1–500 μM). MTT assay was performed to measure the metabolic activity in living cells. The mitochondria succinate dehydrogenase system of living cells metabolized yellow-colored, water-soluble, tetrazolium salt MTT (3-(4,5-dimethylthiazol2-yl)-2,5-diphenyltetrazolium bromide to a purple insoluble formazan crystal. The cells were incubated with the test solutions for 24 h, the MTT solution (10 mg/mL in PBS) was then added to each well, and the mixture was incubated at 37 °C for 3 h. Thereafter, the MTT solution was carefully aspirated, and the obtained formazan crystals were dissolved by the addition of 100 mL DMSO. The cell viability was determined by measuring the optical density −570–690 nm in a multiplate reader Synergy 2 (BioTek Instruments, Inc., Highland Park, Winooski, VT, USA) [50].

#### 4.5.3. H_2_O_2_-Induced Oxidative Stress Model in SH-SY5Y Cells

For the H_2_O_2_-induced oxidative stress model, SH-SY5Y cells were seeded in 96-well plates at a density of 3.5 × 10^4^/well in 100 μL for 24 h. Afterwards, the cell medium was aspirated, and the cells were treated with different concentrations of tested compounds (1, 10, 20 µM) for 90 min; then, the SH-SY5Y cells were washed with phosphate-buffered saline (PBS) and were exposed to the oxidative stress (H_2_O_2_ 1 mM in PBS, 15 min). The contents of all wells were changed with culture medium. After 24 h, the amount of attached viable cells was evaluated by MTT assay. Negative controls (cells without hydrogen peroxide treatment) were considered as 100% protection, and hydrogen-peroxide-treated cells as 0% protection.

#### 4.5.4. Statistical Analysis

Statistical analysis of data has been carried out on GraphPad Prism 6 Software. All experiments were carried out in triplicate, and results were presented as mean ± SD (n = 8). Comparisons between groups have been performed by applying one-way ANOVA with Dunnet’s multiple comparisons post-test. We performed statistical analyses on the data with the aim of validating the significance of the observed differences. Differences between groups were considered significant for *p* < 0.05, *p* < 0.01 and *p* < 0.001.

## 5. Conclusions

Ten new N-pyrrolyl hydrazide–hydrazones were synthesized. The structures of the new compounds were elucidated through appropriate IR, ^1^H-NMR, and MS spectral data. The purity of the compounds was proven by the corresponding melting points, TLC characteristics, and elemental analyses.

The DFT studies have indicated that the best radical scavenging ligands (**7d** and **8d**) in the newly synthesized compounds are stable molecules that do not decompose into elements, are less polarizable, and with hard nature. The energy of HOMO revealed that both compounds possess good electron donation capacities. Overall, **7d** and **8d** can readily scavenge free radicals in the biological system by donation of hydrogen atoms and single electron transfer.

The performed in vitro neurotoxicity and cellular toxicity and cell protection evaluations on human neuroblastoma cell line SH-SY5Y determined **7d** as the most prospective with the lowest toxicity and highest antioxidant protection.

## Figures and Tables

**Table 1:** Reaction conditions, reaction times, and yields.

Reaction Media	Reaction Temperature °C	Reaction Time (min)	Yields %
Ethanol	Heating	50–60	46–80
Ethanol + HCl	Room temperature	20–30	56–94
Methanol + conc. HCl	Room temperature	1440	15–56
Ethanol + glacial acetic acid	100 °C	30–50	26–64
**Glacial acetic acid**	**100 °C**	**20–30**	**68–84**

**Table 2:** IDs, melting points, TLC characteristics (Rf), MS data, and yields for the new N-pyrrolyl-hydrazones.

IDs	m.p. °C	Rf	MS Data [M+H]^+^ (*m/z*)	Yields %
**7a**	211.4–213.6	0.38	511.13	84
**7b**	229.4–231.2	0.33	502.05	78
**7c**	245.9–247.2	0.33	512.07	76
**7d**	191.9–194.4	0.29	544.10	68
**7e**	206.0–207.6	0.40	528.11	72
**8a**	212.0–213.3	0.35	525.15	86
**8b**	181.4–184.6	0.31	517.06	74
**8c**	214.4–217.1	0.33	527.09	82
**8d**	196.6–197.6	0.28	558.12	64
**8e**	170.0–171.9	0.34	542.13	74

**Table 3:** FMOs energies and global reactivity descriptors for **7d** and **8d** at B3LYP/6–311++(d,p) level of theory.

Electronic Parameter	7d	8d
E_HOMO_	−0.2013	−0.1976
E_LUMO_	−0.0564	−0.0470
ΔE_HOMO−LUMO_	0.1449	0.1506
Ionization Energy (IP)	0.2013	0.1976
Electron Affinity	0.0564	0.0470
Chemical Hardness	0.0724	0.0753
Softness	6.9060	6.6401
Electronegativity	0.1288	0.1223
Chemical Potential	−0.1288	−0.1223
Electrophilicity Index	0.1145	0.0992

**Table 4:** Calculated bond dissociation energies (BDEs) and ionization potentials (IPs) of the corresponding compounds in gas phase.

Compound	Bond	BDE (Kcal/mol)	IP (Kcal/mol)
**7d**	O_31_-H	83.55	
	C_7_-H	90.4	126.31
	N_22_-H	95.10	
**8d**	O_30_-H	83.09	
	C_7_-H	88.9	123.99
	N_21_-H	96.2	

**Table 5:** In vitro cytotoxicity evaluation of the newly synthesized derivatives **7a**–**e** and **8a**–**e** on SH-SY5Y cells (IC50 values).

Compound IDs	IC50 [µM]	95% Confidence Intervals (CI)
**7a**	55.75	55.65–61.37
**7b**	67.60	54.31–79.64
**7c**	>500	NA
**7d**	99.56	88.87–103.23
**7e**	63.08	55.56–73.23
**8a**	56.33	45.25–67.63
**8b**	57.36	46.26–68.36
**8c**	58.23	47.36–69.23
**8d**	57.26	46.29–68.39
**8e**	91.07	83.65–105-36
Melatonin	>500	NA

## Data Availability

Data is contained within the article and supplementary material.

## Supplementary Materials

The following supporting information can be downloaded at https://www.mdpi.com/article/10.3390/ph16091198/s1.
