# Supplementary material for: Synthesis, DFT Study, and In Vitro Evaluation of Antioxidant Properties and Cytotoxic and Cytoprotective Effects of New Hydrazones on SH-SY5Y Neuroblastoma Cell Lines
Source: Pharmaceuticals (Basel). 2023 Aug 23;16(9):1198. doi: 10.3390/ph16091198 (PMC10537553; doi:10.3390/ph16091198)
Supplement: Supplementary file 1 [file pharmaceuticals-16-01198-s001.zip › pharmaceuticals-2557951-supplementary.pdf]

Article

Synthesis, DFT Study, and In Vitro Evaluation of Antioxidant Properties and Cytotoxic and Cytoprotective Effects of New Hydrazones on SH-SY5Y Neuroblastoma Cell Lines

Diana Tzankova [1], Hristina Kuteva [2], Emilio Mateev [1], Denitsa Stefanova [2], Alime Dzhemadan [2], Yordan Yordanov [2], Alexandrina Mateeva [1], Virginia Tzankova [2], Magdalena Kondeva-Burdina [2], Alexander Zlatkov [1] and Maya Georgieva [1,*]

1 Department "Pharmaceutical Chemistry", Faculty of Pharmacy, Medical University-Sofia, 2 Dunav Str., 1000 Sofia, Bulgaria; d.tsankova@pharmfac.mu-sofia.bg (D.T.); e.mateev@pharmfac.mu-sofia.bg (E.M.); a.dineva@pharmfac.mu-sofia.bg (A.M.); azlatkov@pharmfac.mu-sofia.bg (A.Z.)

2 Laboratory "Drug metabolism and Drug Toxicity", Department "Pharmacology, Pharmacotherapy and Toxicology", Faculty of Pharmacy, Medical University-Sofia, 2 Dunav Str., 1000 Sofia, Bulgaria; hristina.kuteva@abv.bg (H.K.); denitsa.stefanova@pharmfac.mu-sofia.bg (D.S.); yyordanov@pharmfac.mu-sofia.bg (Y.Y.); vtzankova@pharmfac.mu-sofia.bg (V.T.); mkondeva@pharmfac.mu-sofia.bg (M.K.-B.)

* Correspondence: mgeorgieva@pharmfac.mu-sofia.bg

Supplementary Materials

The IR, ^{1}H NMR and MS characterization spectra of the synthesized compounds.

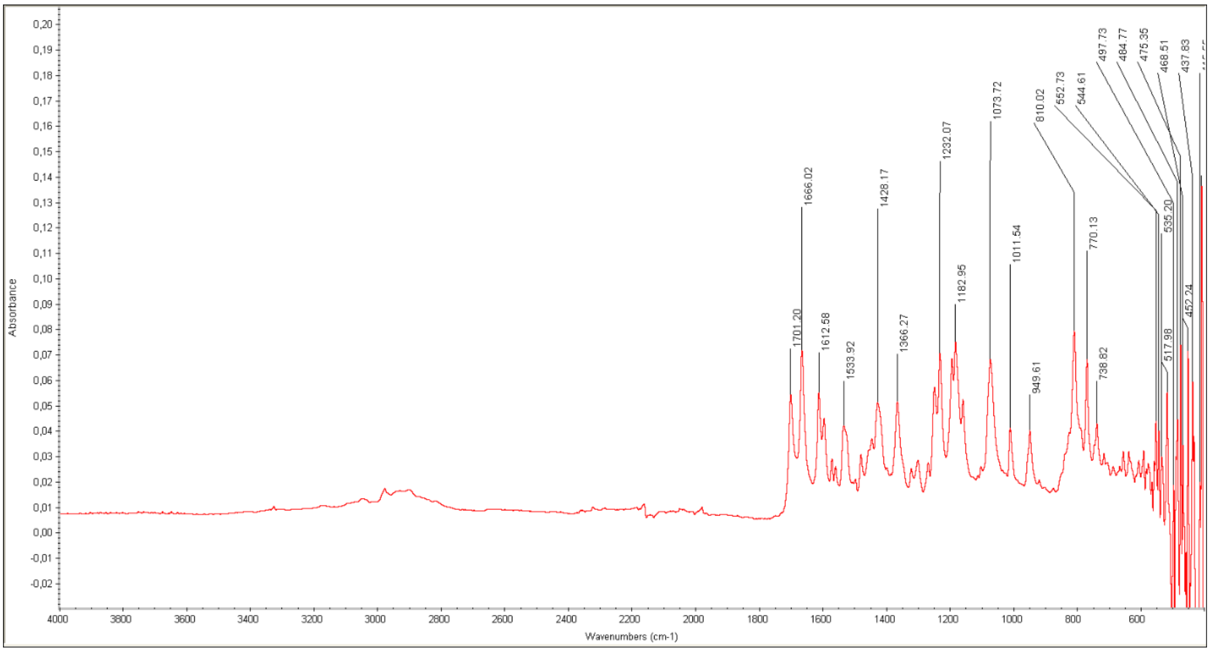

Figure S1. IR spectrum of ethyl 5-(4-bromophenyl)-1-(2-(2-(4-(dimethylamino) benzylidene)hydrazinyl)-2-oxoethyl)-2-methyl-1H-pyrrole-3-carboxylate (7a).

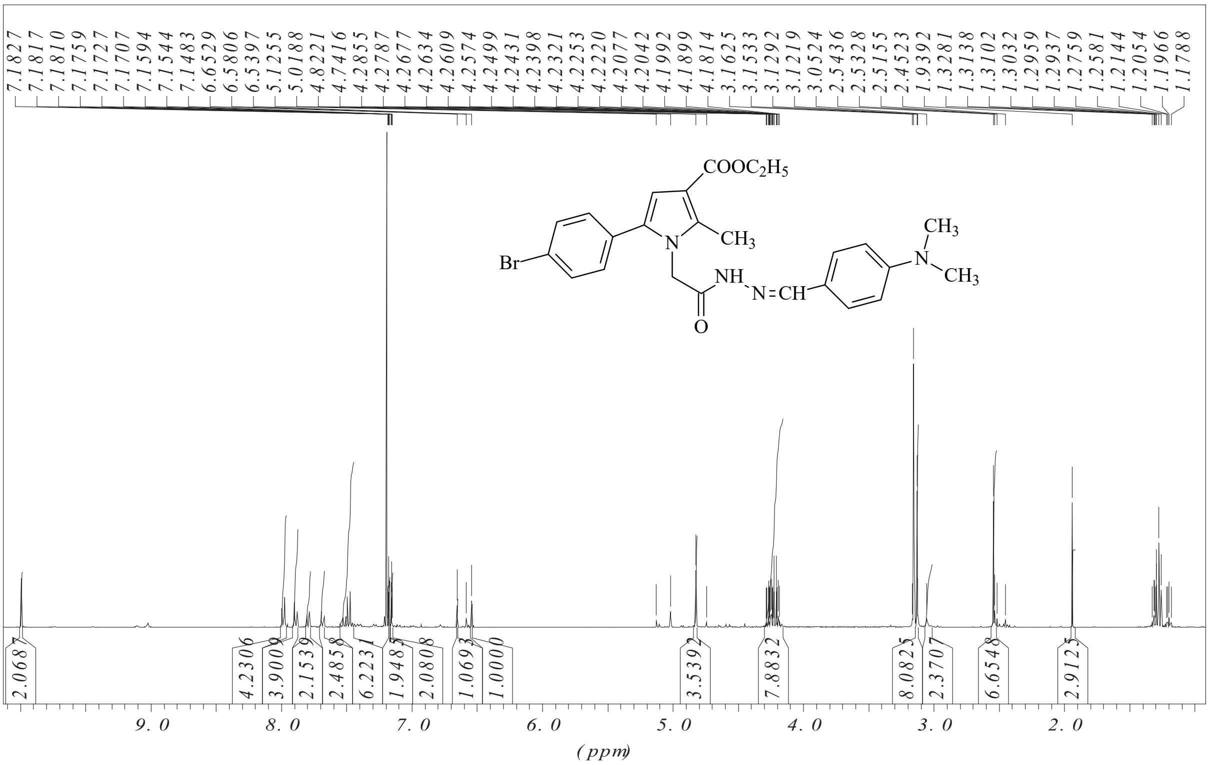

Figure S2. ^{1}H-NMR spectrum ($CDCl_3$, 250 MHz) of compound 7a.

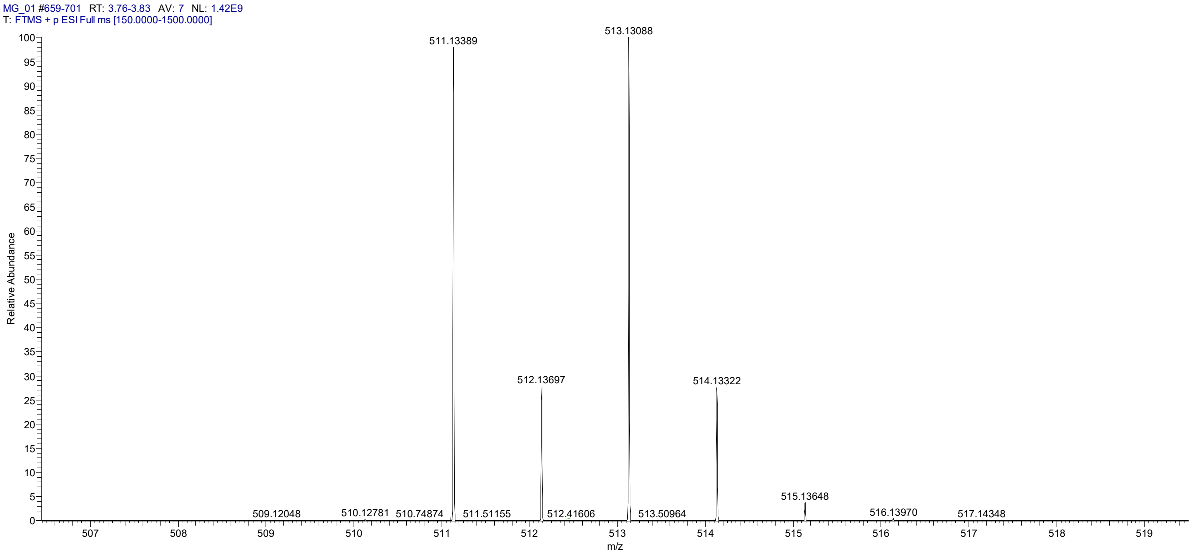

Figure S3. MS spectrum (ESI) of ethyl 5-(4-bromophenyl)-1-(2-(2-(4-(dimethylamino) benzylidene)hydrazinyl)-2-oxoethyl)-2-methyl-1H-pyrrole-3-carboxylate (7a).

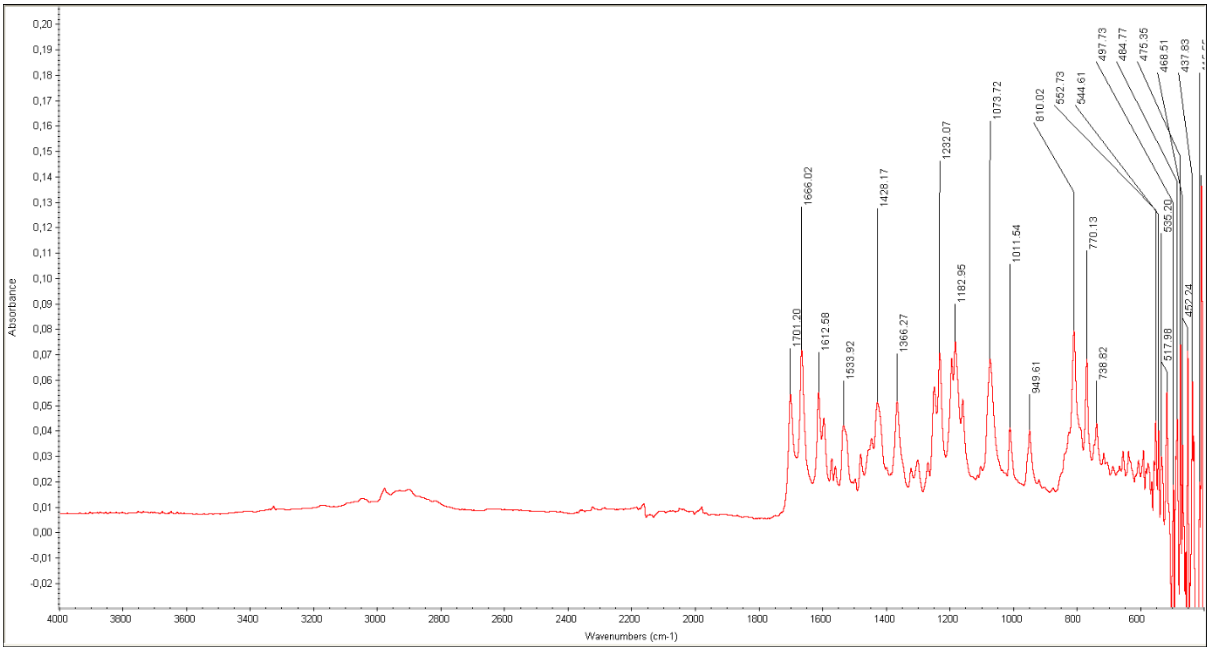

Figure S4. IR spectrum of ethyl 5-(4-bromophenyl)-1-(2-(2-(4-chlorobenzylidene) hydrazinyl)-2-oxoethyl)-2-methyl-1H-pyrrole-3-carboxylate (7b).

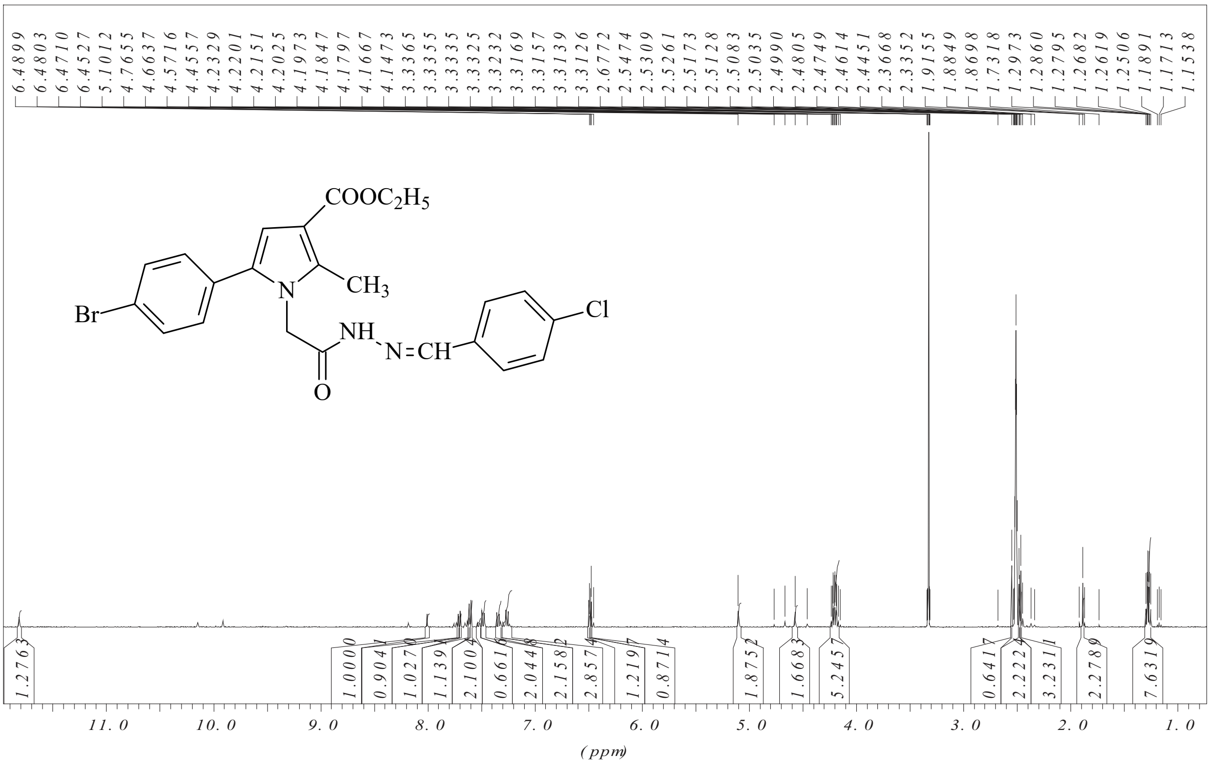

Figure S5. ^{1}H-NMR spectrum (CDCl$_3$, 250 MHz) of compound 7b.

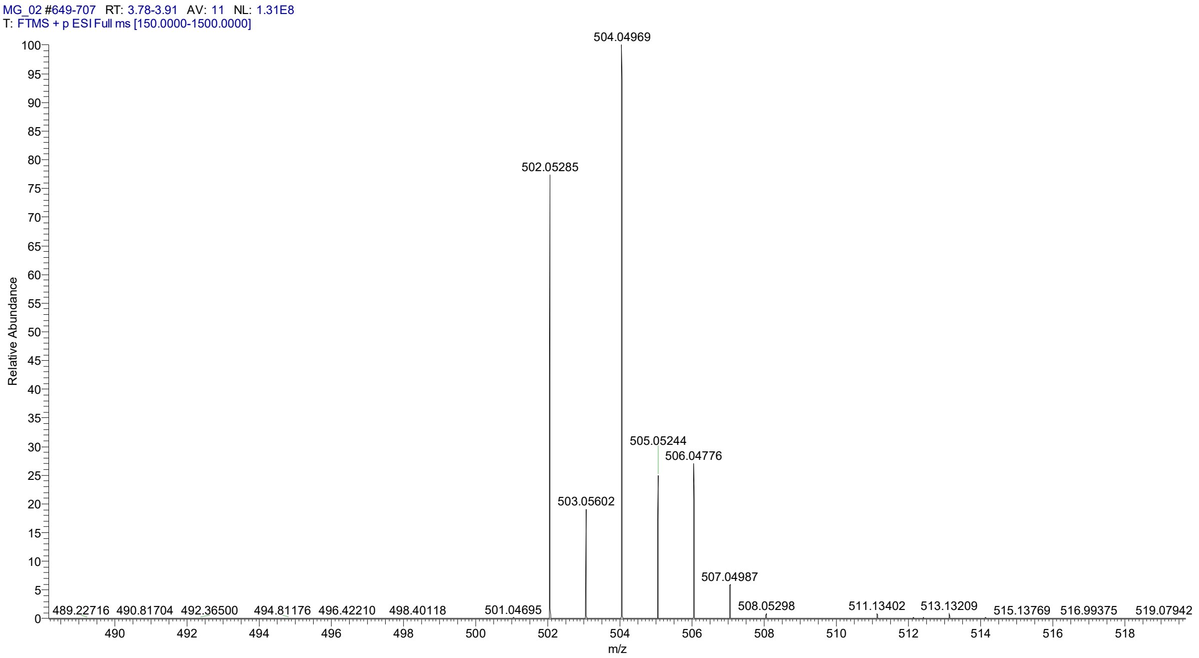

Figure S6. MS spectrum (ESI) of ethyl 5-(4-bromophenyl)-1-(2-(2-(4-chlorobenzylidene) hydrazinyl)-2-oxoethyl)-2-methyl-1H-pyrrole-3-carboxylate (7b).

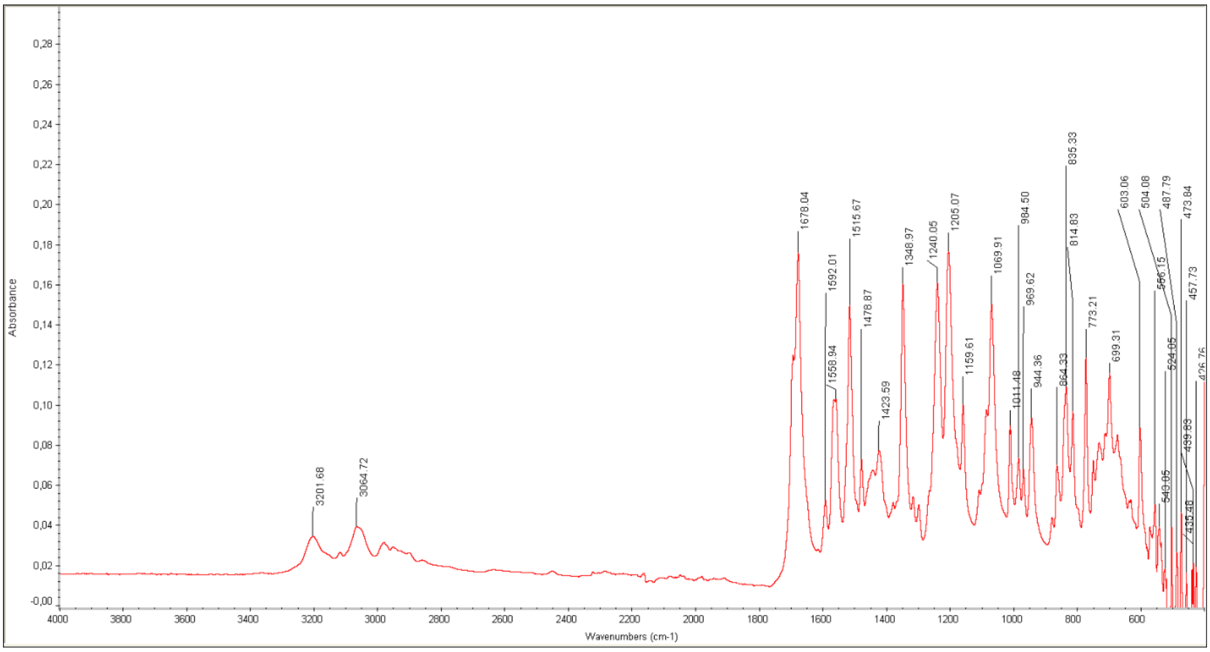

Figure S7. IR spectrum of ethyl 5-(4-bromophenyl)-2-methyl-1-(2-(2-(4-nitrobenzylidene) hydrazinyl)-2-oxo-ethyl)-1H-pyrrole-3-carboxylate (7c).

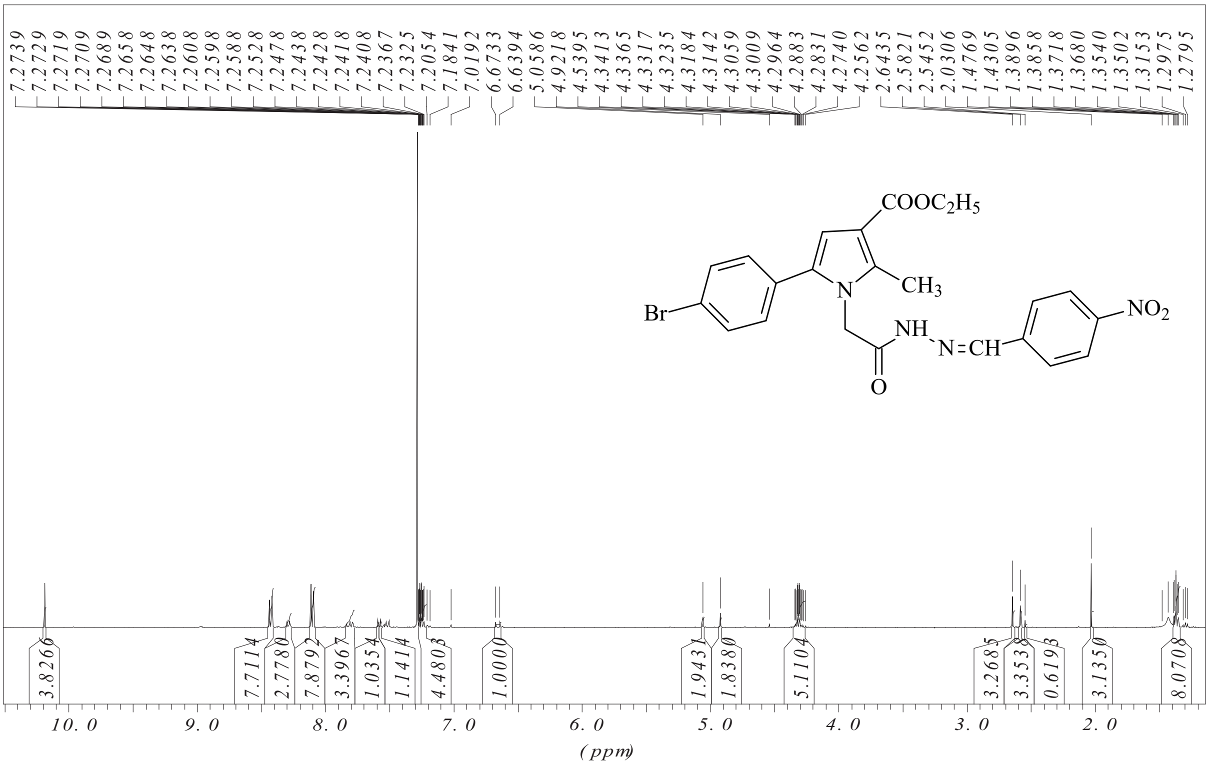

Figure S8. ^{1}H-NMR spectrum ($CDCl_3$, 250 MHz) of compound 7c.

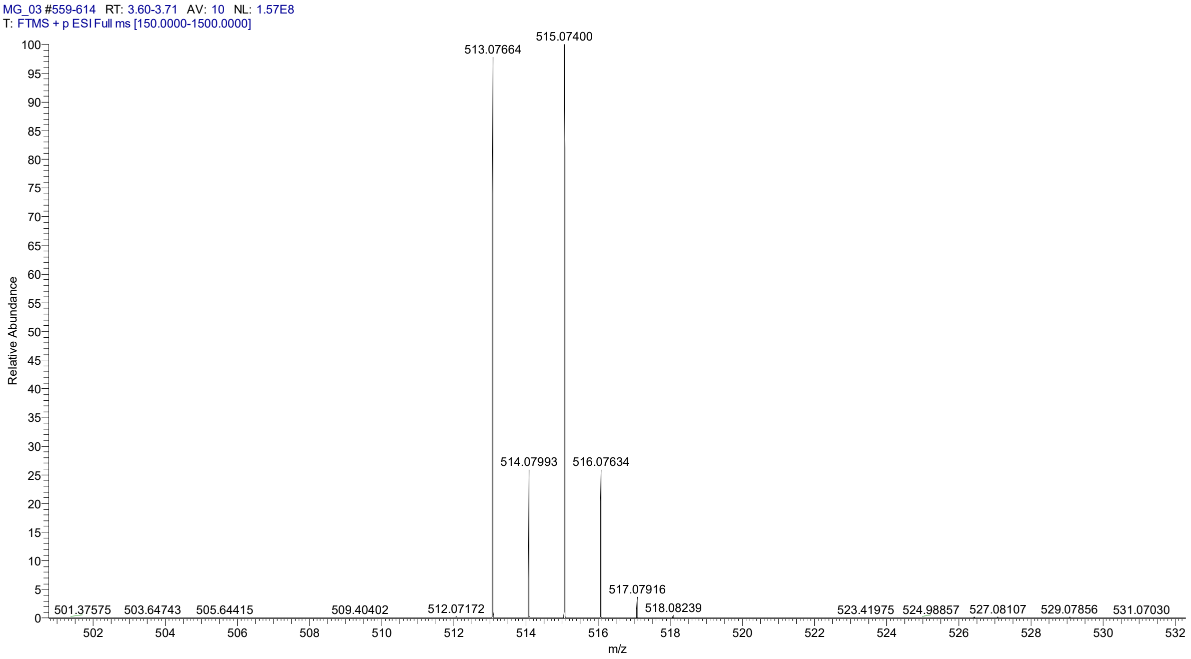

Figure S9. MS spectrum (ESI) of ethyl 5-(4-bromophenyl)-2-methyl-1-(2-(2-(4-nitrobenzylidene) hydrazinyl)-2-oxo-ethyl)-1H-pyrrole-3-carboxylate (7c).

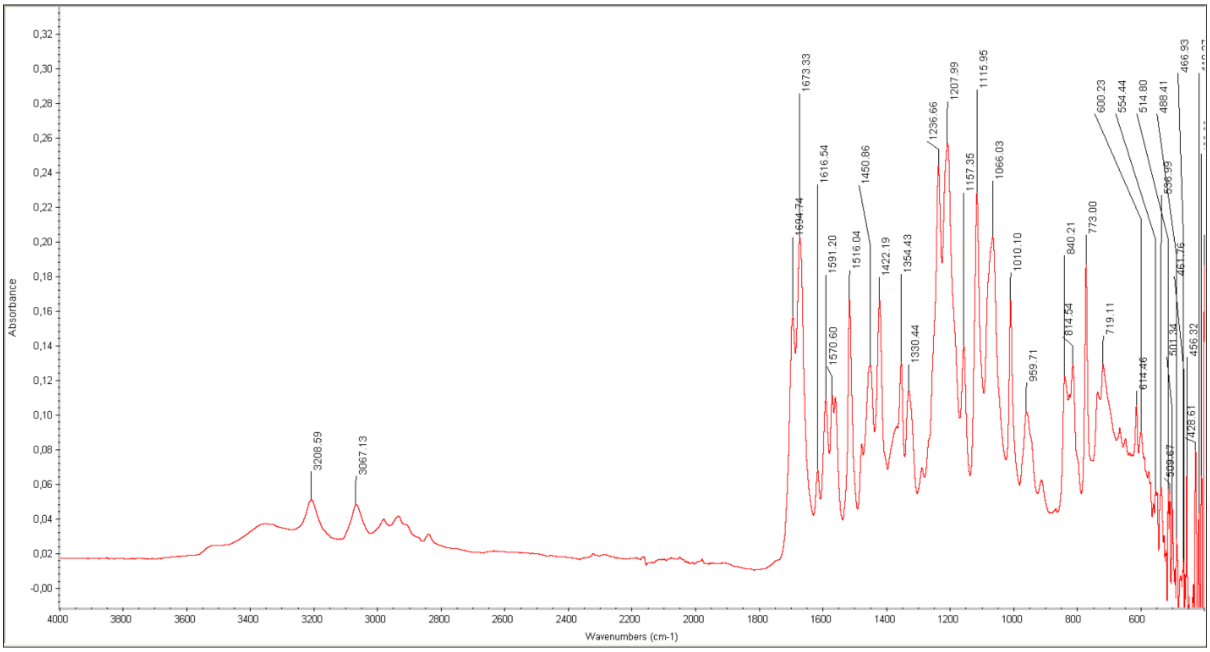

Figure S10. IR spectrum of ethyl 5-(4-bromophenyl)-1-(2-(2-(4-hydroxy-3,5-dimethoxybenzylidene)hydrazine-yl)-2-oxoethyl)-2-methyl-1H-pyrrole-3-carboxylate (7d).

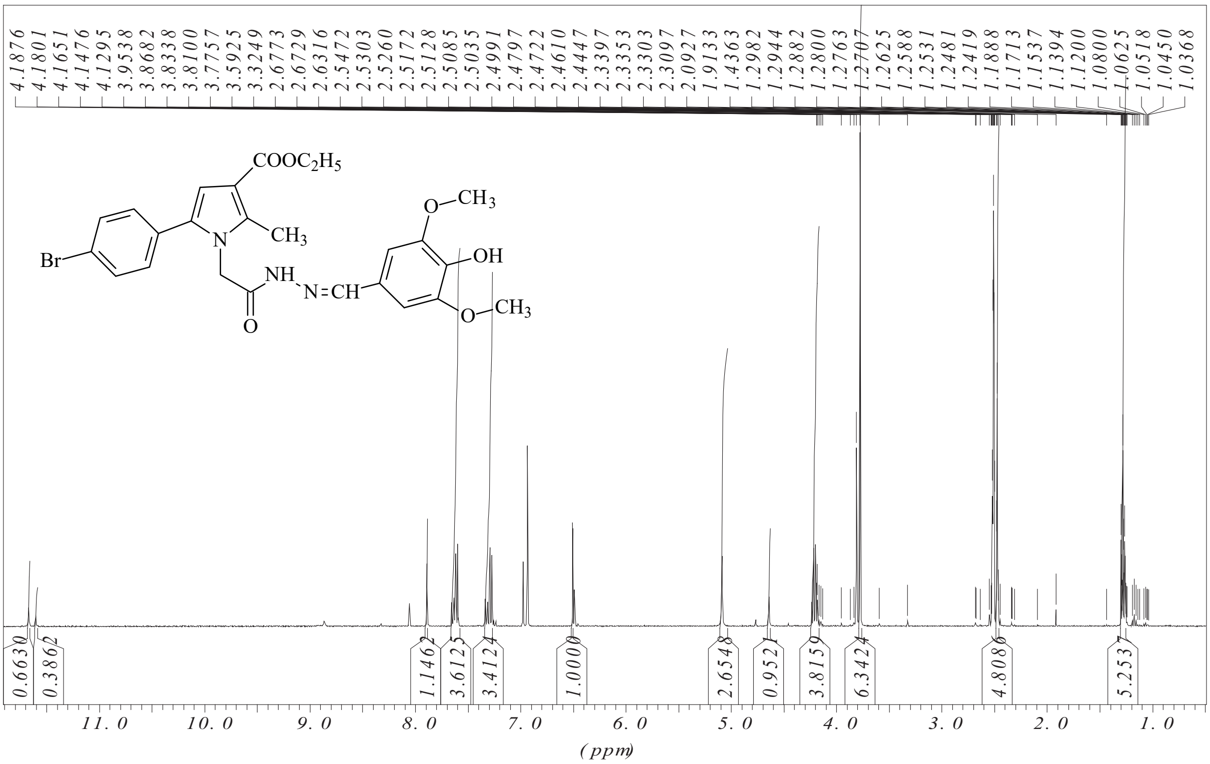

Figure S11. ^{1}H-NMR spectrum ($CDCl_3$, 250 MHz) of compound 7d.

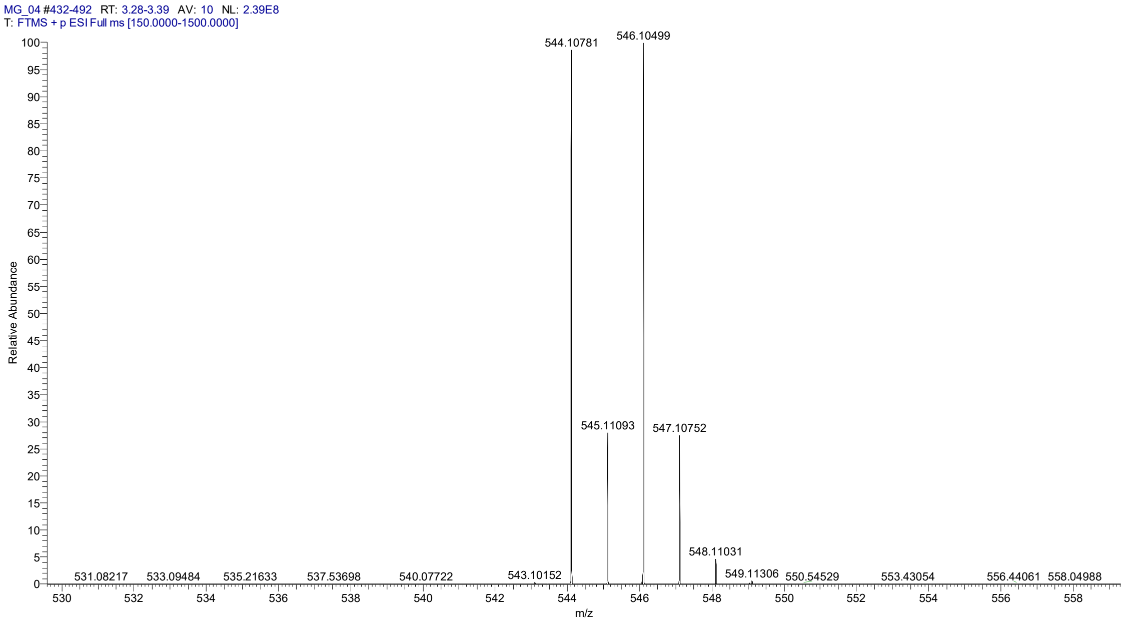

Figure S12. MS spectrum (ESI) of ethyl 5-(4-bromophenyl)-1-(2-(2-(4-hydroxy-3,5-dimethoxybenzylidene)hydrazine-yl)-2-oxoethyl)-2-methyl-1H-pyrrole-3-carboxylate (7d).

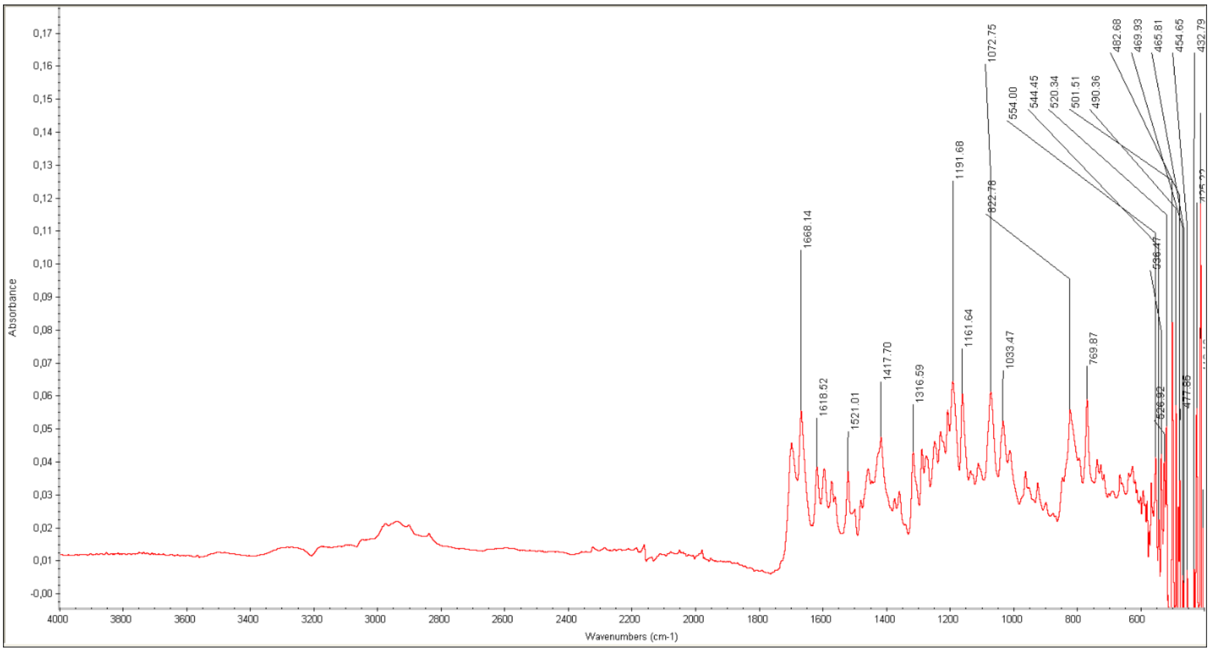

Figure S13. IR spectrum of ethyl 5-(4-bromophenyl)-1-(2-(2-(2,4-dimethoxybenzylidene) hydrazinyl)-2-oxo-ethyl)-2-methyl-1H-pyrrole-3-carboxylate (7e).

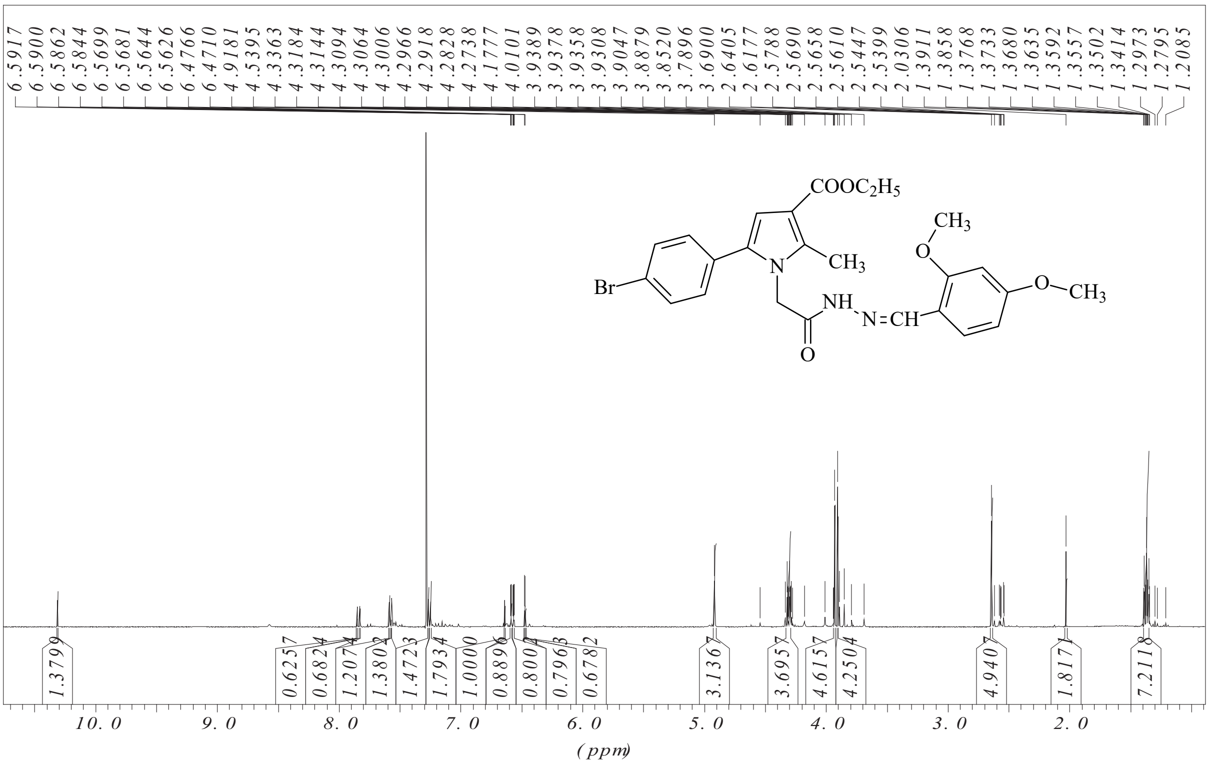

Figure S14. ^{1}H-NMR spectrum ($CDCl_3$, 250 MHz) of compound 7e.

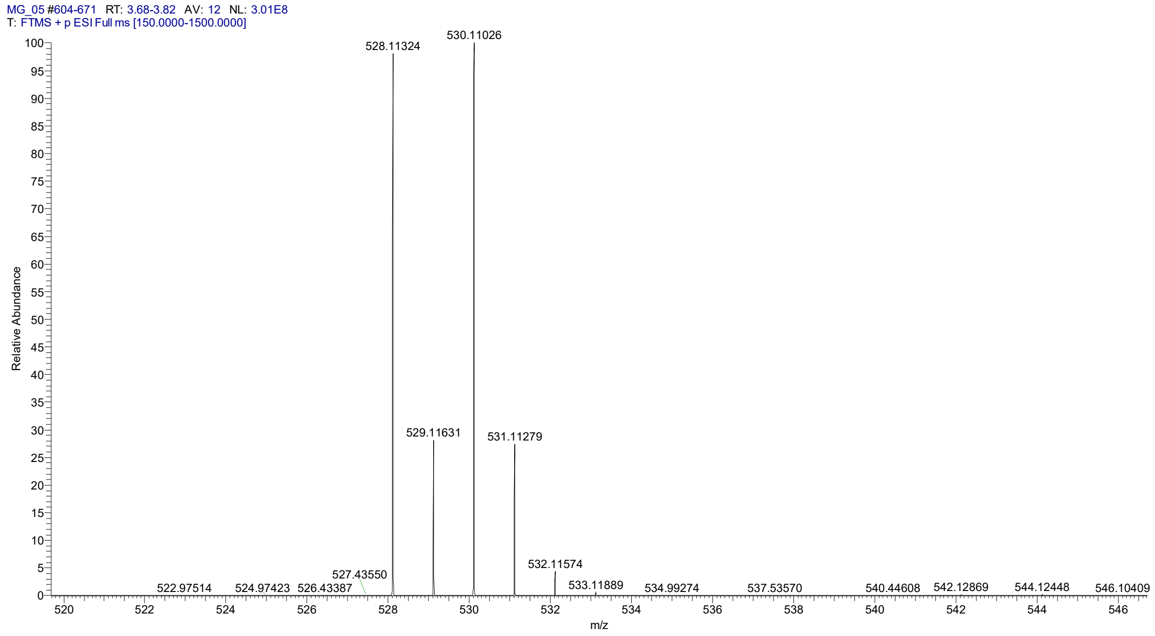

Figure S15. MS spectrum (ESI) of ethyl 5-(4-bromophenyl)-1-(2-(2-(2,4-dimethoxybenzylidene)hydrazinyl)-2-oxo-ethyl)-2-methyl-1H-pyrrole-3-carboxylate (7e).

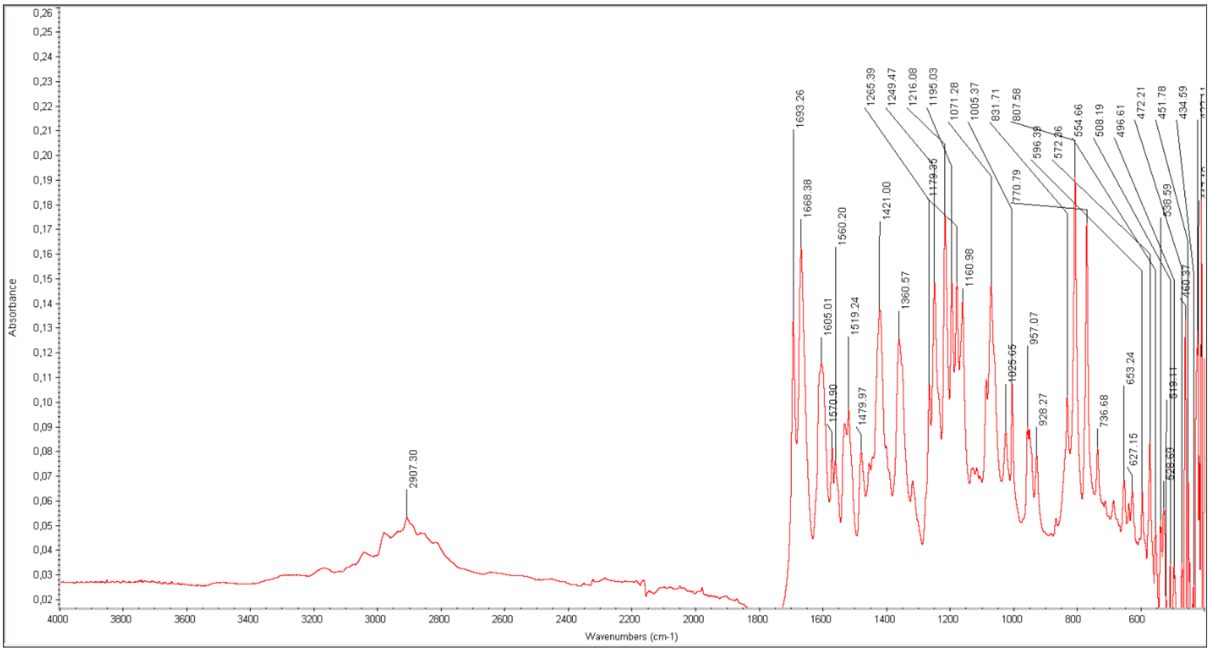

Figure S16. IR spectrum of ethyl 5-(4-bromophenyl)-1-(3-(2-(4-(dimethylamino) benzylidene)hydrazinyl)-3-oxopropyl)-2-methyl-1H-pyrrole-3-carboxylate (8a).

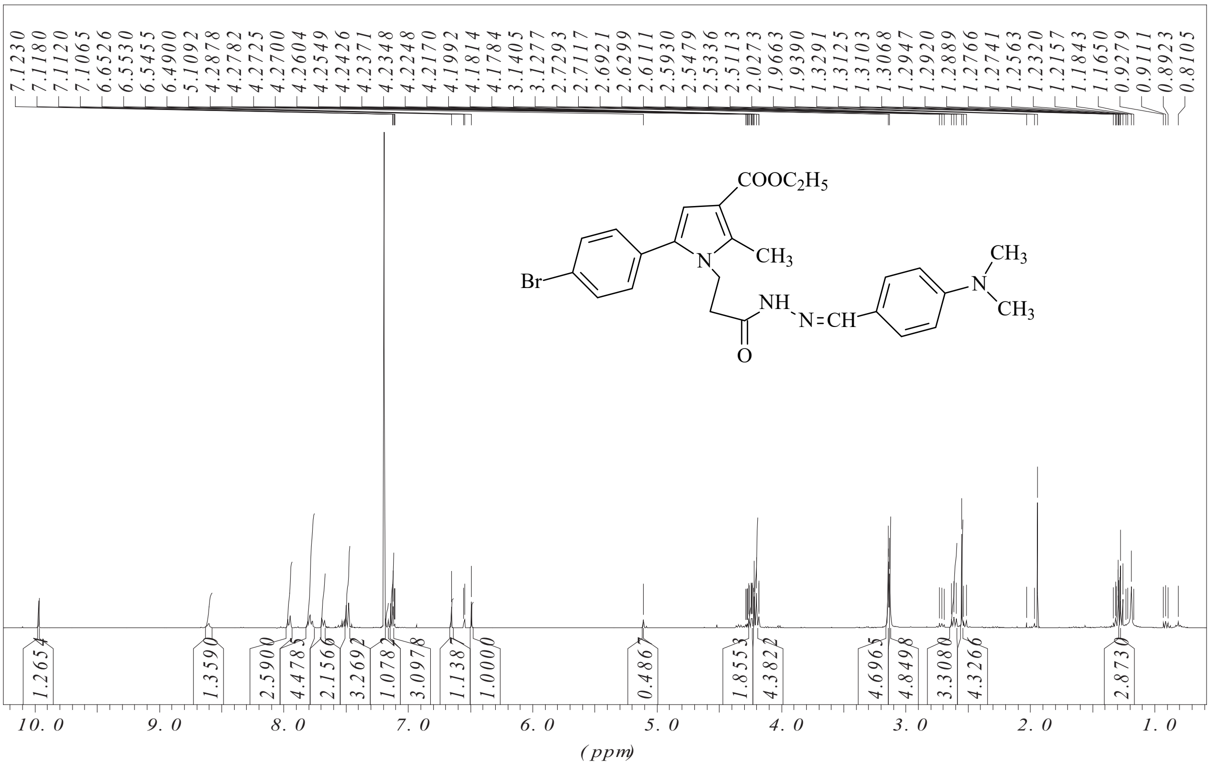

Figure S17. ^{1}H-NMR spectrum ($CDCl_3$, 250 MHz) of compound 8a.

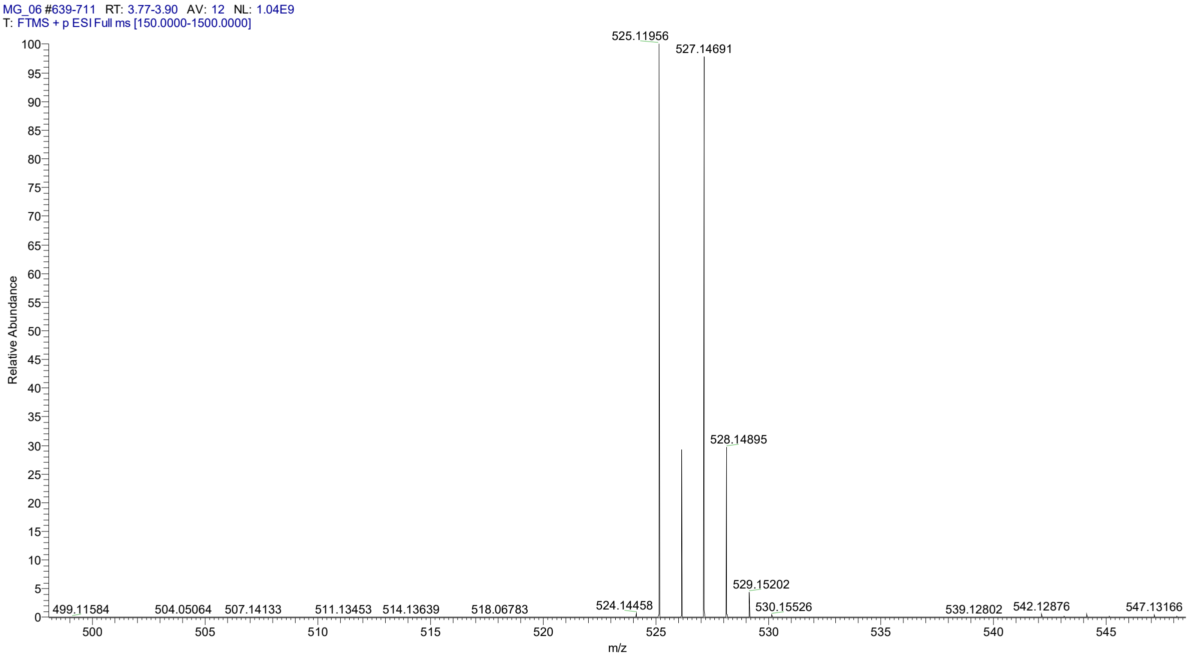

Figure S18. MS spectrum (ESI) of ethyl 5-(4-bromophenyl)-1-(3-(2-(4-(dimethylamino) benzylidene)hydrazinyl)-3-oxopropyl)-2-methyl-1H-pyrrole-3-carboxylate (8a).

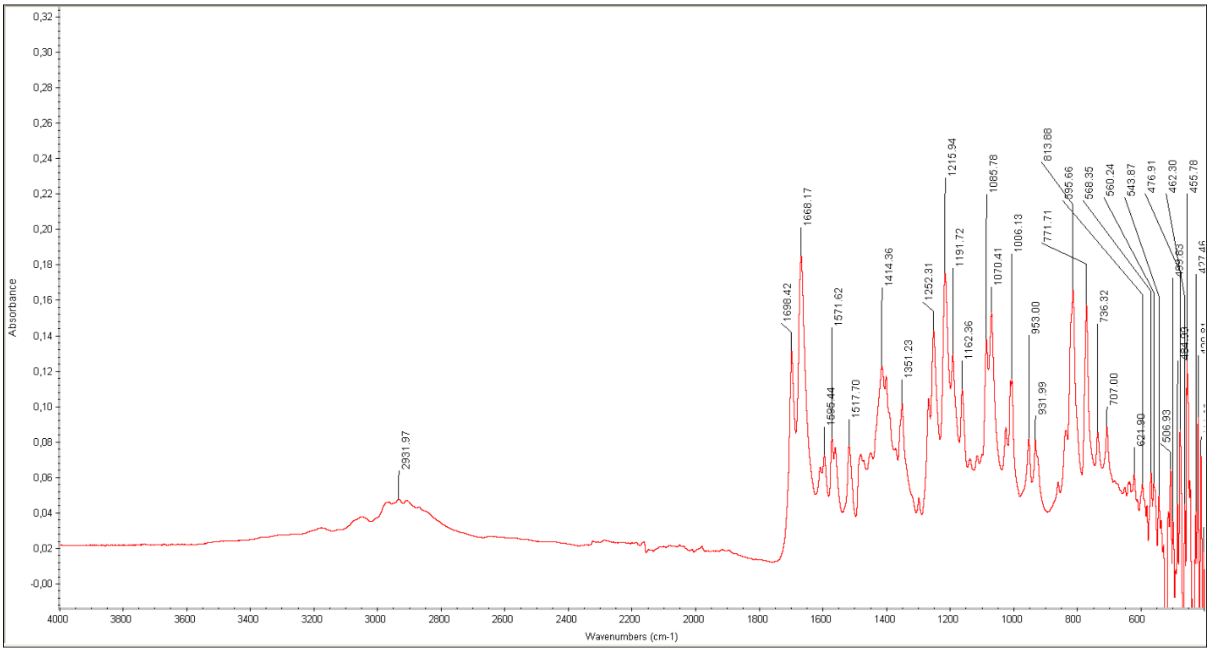

Figure S19. IR spectrum of ethyl 5-(4-bromophenyl)-1-(3-(2-(4-chlorobenzylidene) hydrazinyl)-3-oxopropyl)-2-methyl-1H-pyrrole-3-carboxylate (8b).

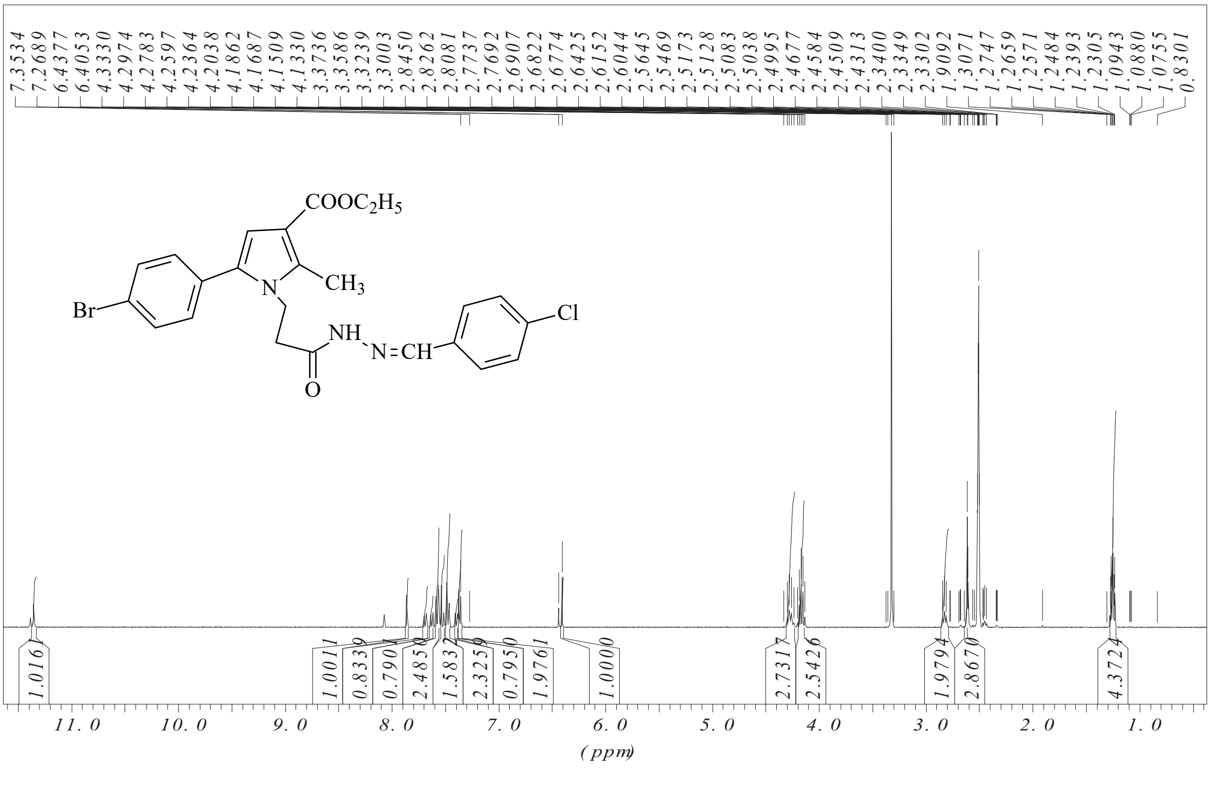

Figure S20. ^{1}H-NMR spectrum (CDCl$_3$, 250 MHz) of compound 8b.

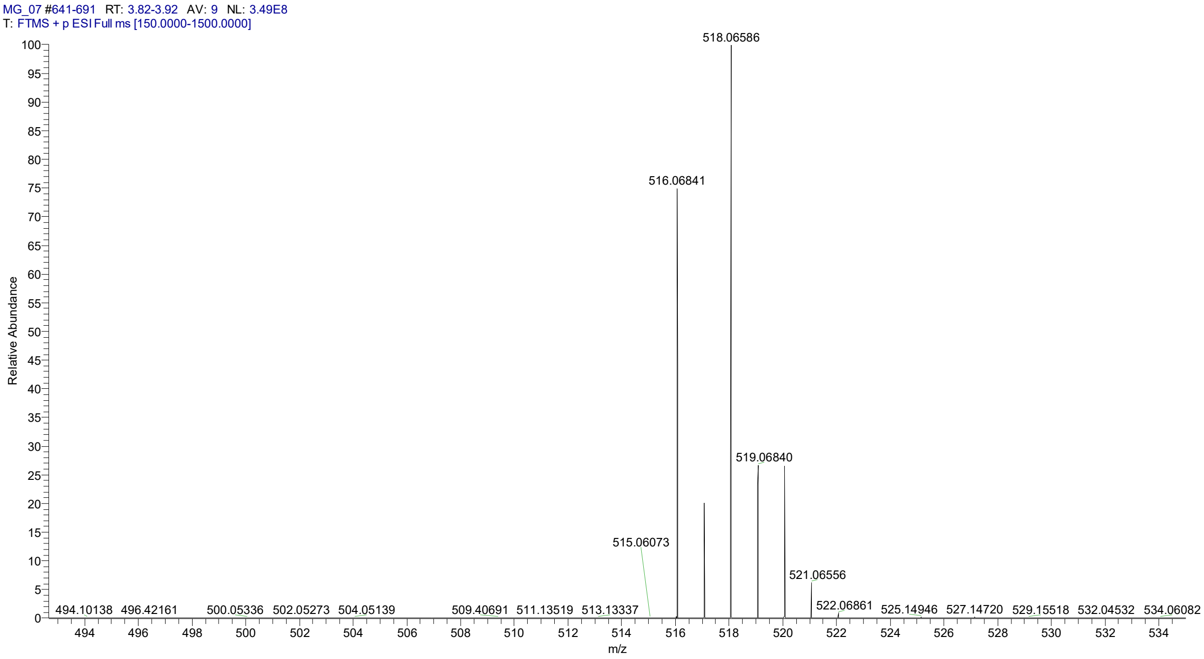

Figure S21. MS spectrum (ESI) of ethyl 5-(4-bromophenyl)-1-(3-(2-(4-chlorobenzylidene) hydrazinyl)-3-oxopropyl)-2-methyl-1H-pyrrole-3-carboxylate (8b).

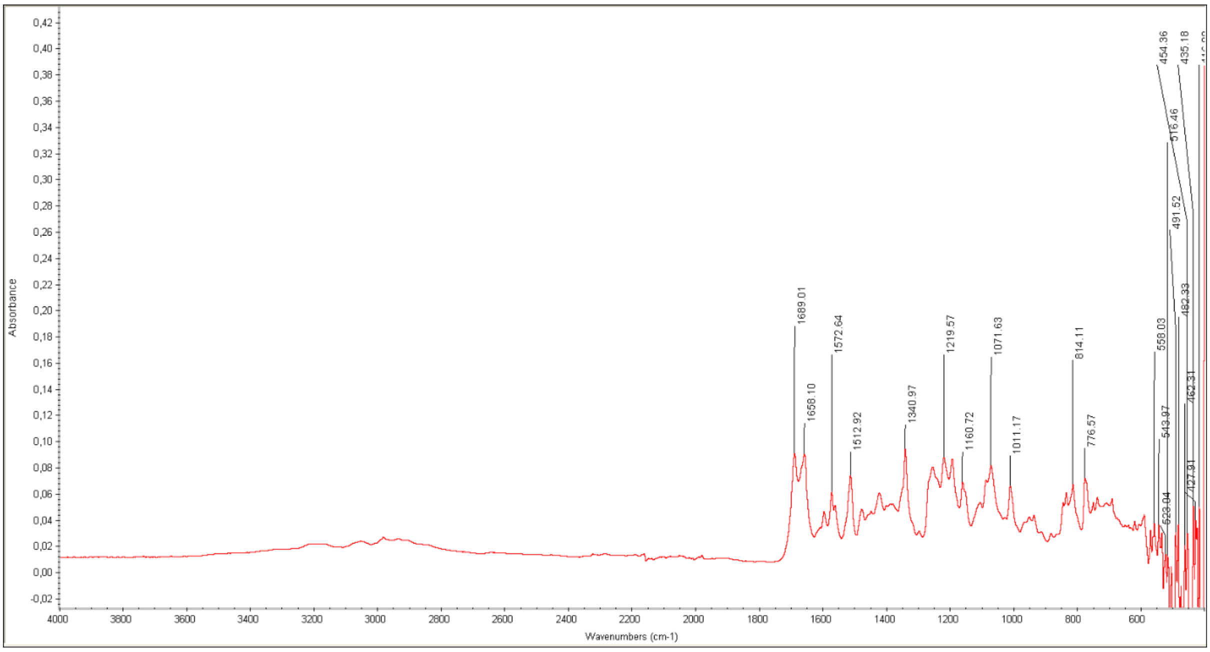

Figure S22. IR spectrum of ethyl 5-(4-bromophenyl)-2-methyl-1-(3-(2-(4-nitrobenzylidene) hydrazinyl)-3-oxo-propyl)-1H-pyrrole-3-carboxylate (8c).

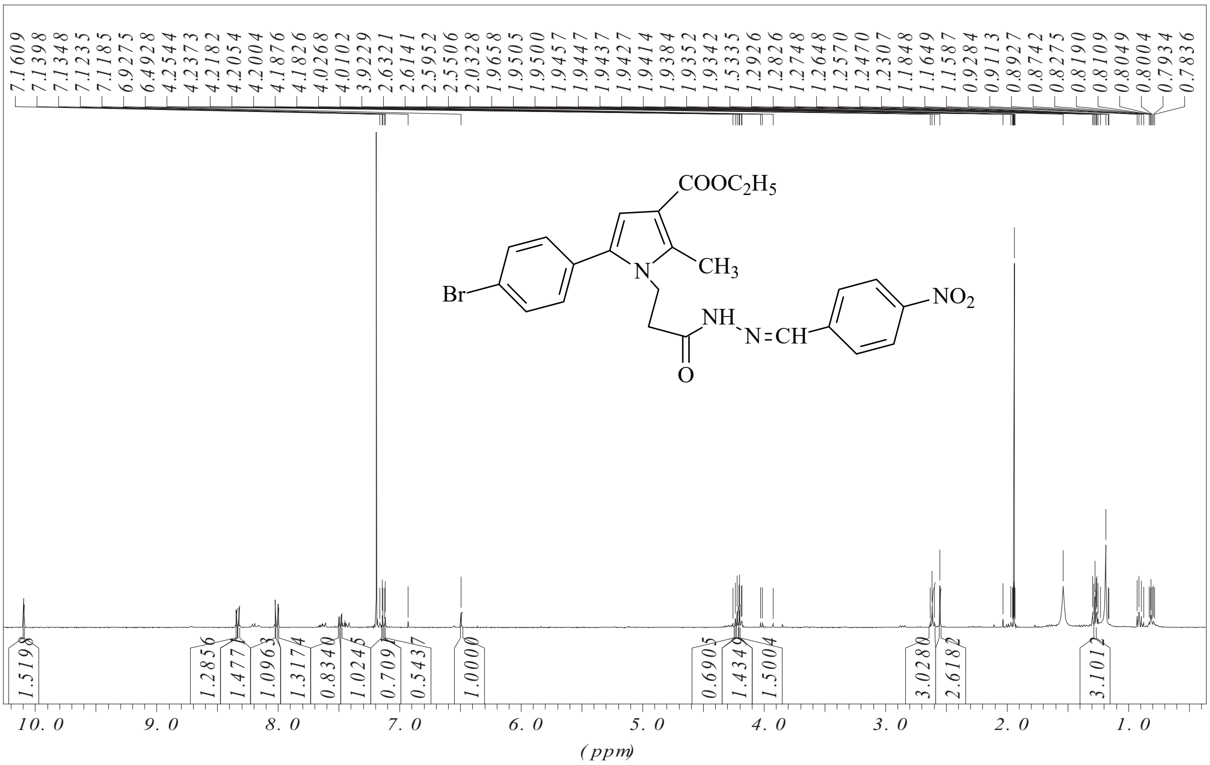

Figure S23. ^{1}H-NMR spectrum (CDCl$_3$, 250 MHz) of compound 8c.

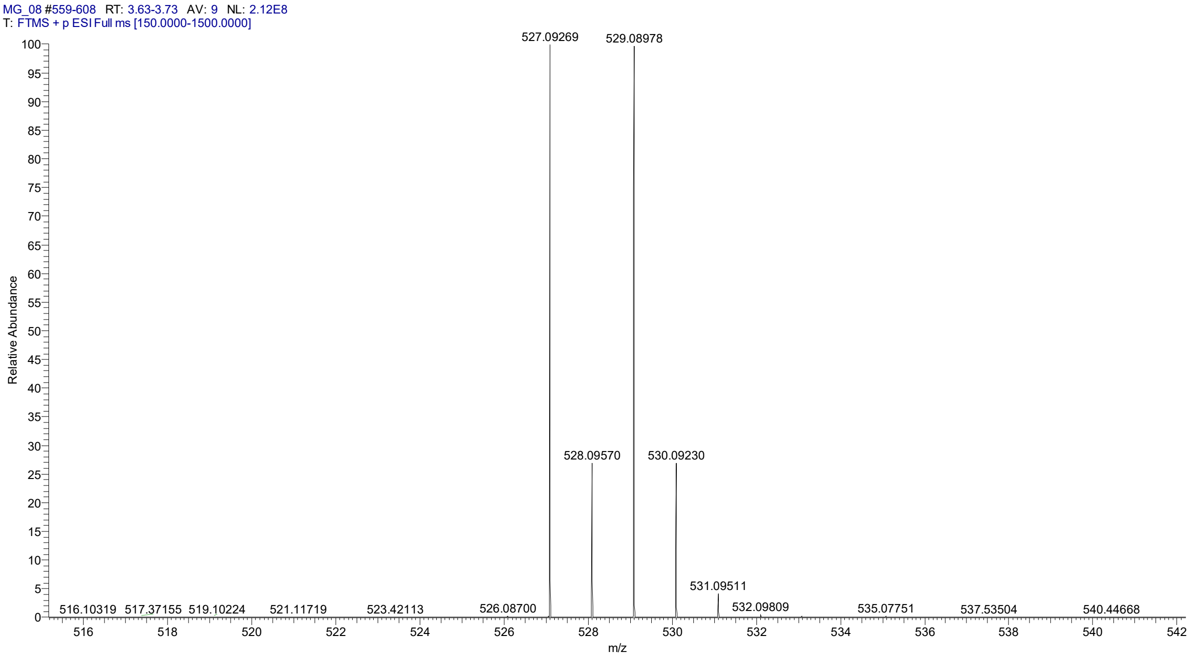

Figure S24. MS spectrum (ESI) of ethyl 5-(4-bromophenyl)-2-methyl-1-(3-(2-(4-nitrobenzylidene)hydrazinyl)-3-oxo-propyl)-1H-pyrrole-3-carboxylate (8c).

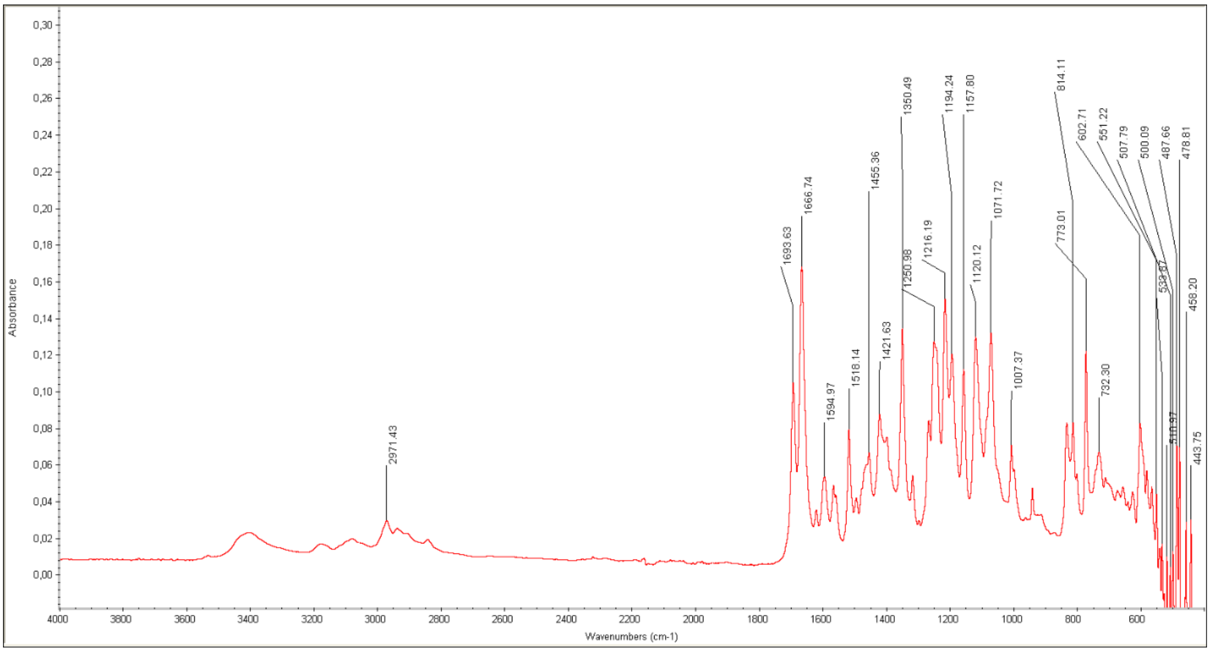

Figure S25. IR spectrum of ethyl 5-(4-bromophenyl)-1-(3-(2-(4-hydroxy-3,5-dimethoxy benzylidene)hydrazine-yl)-3-oxopropyl)-2-methyl-1H-pyrrole-3-carboxylate (8d).

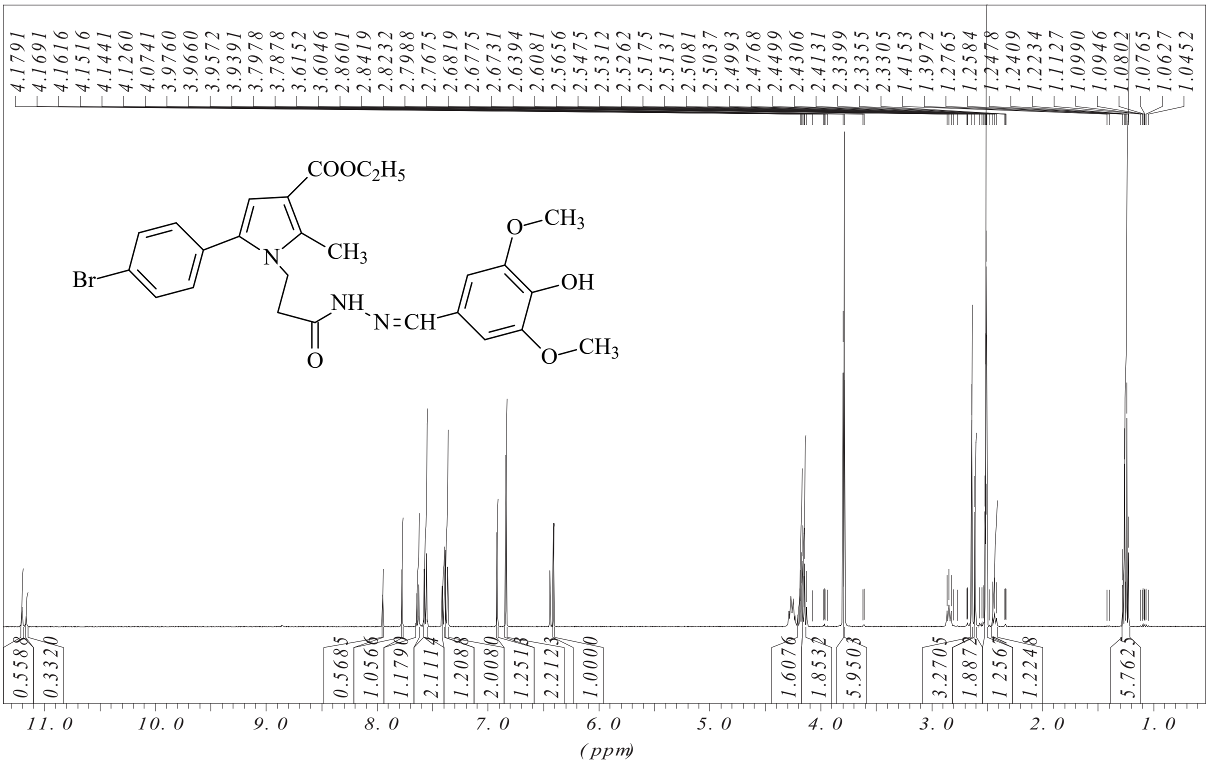

Figure S26. ^{1}H-NMR spectrum ($CDCl_3$,600MHz) of compound 8d.

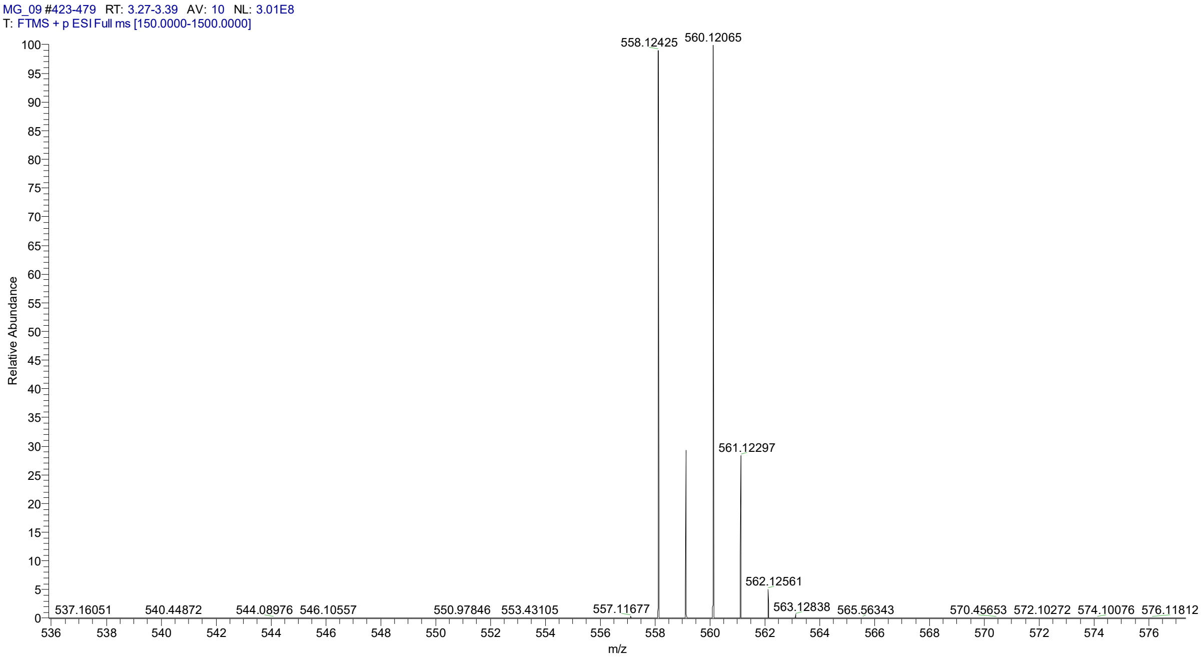

Figure S27. MS spectrum (ESI) of ethyl 5-(4-bromophenyl)-1-(3-(2-(4-hydroxy-3,5-dimethoxybenzylidene)hydrazine-yl)-3-oxopropyl)-2-methyl-1H-pyrrole-3-carboxylate (8d).

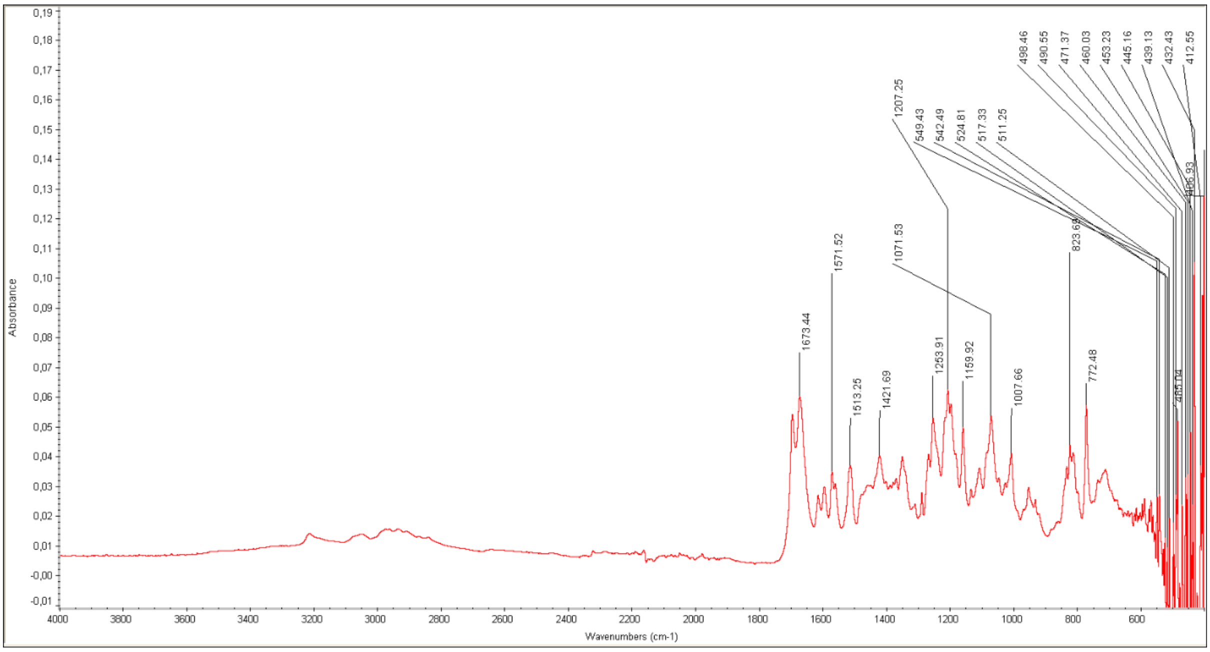

Figure S28. IR spectrum of ethyl 5-(4-bromophenyl)-1-(3-(2-(2,4-dimethoxybenzylidene) hydrazinyl)-3-oxo-propyl)-2-methyl-1H-pyrrole-3-carboxylate (8e).

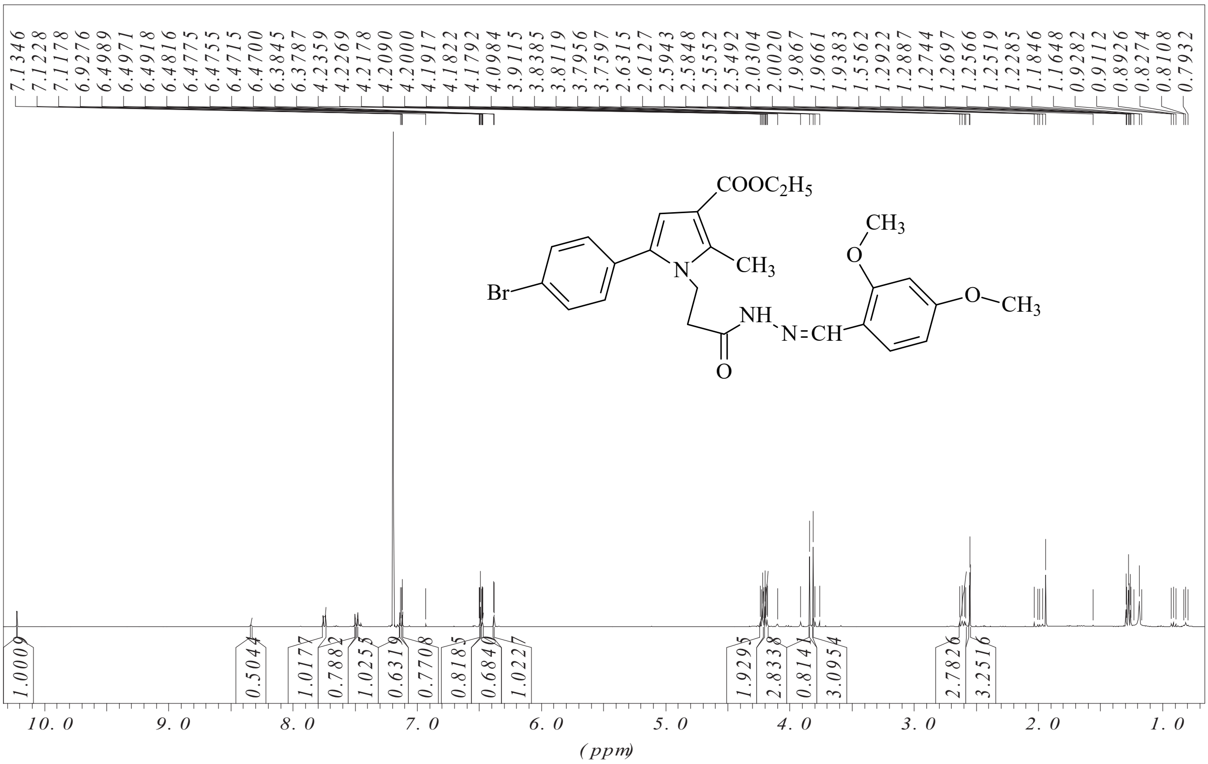

Figure S29. ¹H-NMR spectrum (CDCl₃,600MHz) of compound 8e.

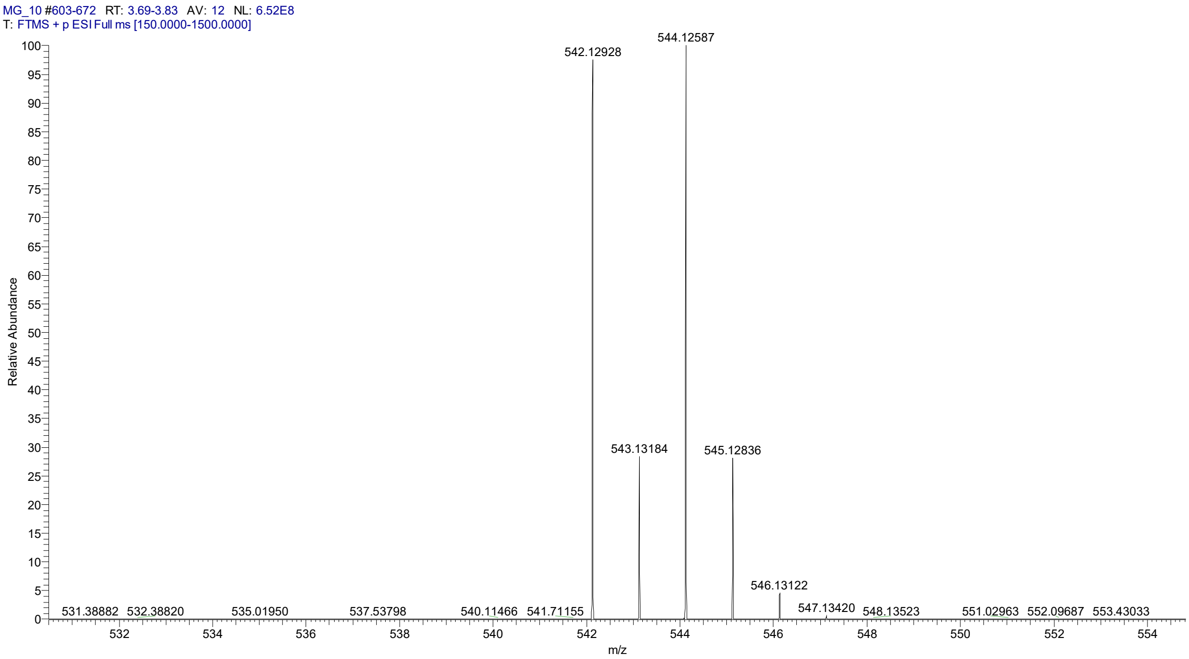

Figure S30. MS spectrum (ESI) of ethyl 5-(4-bromophenyl)-1-(3-(2-(2,4-dimethoxy benzylidene)hydrazinyl)-3-oxo-propyl)-2-methyl-1H-pyrrole-3-carboxylate (8e).
